# Identification and Functional Characterization of Two Major Loci Associated with Resistance against Brown Planthoppers (*Nilaparvata lugens (Stål)*) Derived from *Oryza nivara*

**DOI:** 10.3390/genes14112066

**Published:** 2023-11-11

**Authors:** Akanksha Srivastava, Madhu Pusuluri, Divya Balakrishnan, Jhansi Lakshmi Vattikuti, Sarla Neelamraju, Raman Meenakshi Sundaram, Satendra Kumar Mangrauthia, Tilathoo Ram

**Affiliations:** 1 ICAR-Indian Institute of Rice Research, Hyderabad 500030, India; akanksha.srivastav007@gmail.com (A.S.); madhupusuluri@gmail.com (M.P.); divyab0005@gmail.com (D.B.); rms_28@rediffmail.com (R.M.S.); 2 International Crops Research Institute for the Semi-Arid Tropics (ICRISAT), Hyderabad 502324, India

**Keywords:** brown planthopper (BPH), rice, *Oryza nivara*, *STPKR* gene

## Abstract

The brown planthopper (BPH) is a highly destructive pest of rice, causing significant economic losses in various regions of South and Southeast Asia. Researchers have made promising strides in developing resistance against BPH in rice. Introgression line RPBio4918-230S, derived from *Oryza nivara*, has shown consistent resistance to BPH at both the seedling and adult stages of rice plants. Segregation analysis has revealed that this resistance is governed by two recessive loci, known as *bph39(t)* and *bph40(t)*, contributing to 21% and 22% of the phenotypic variance, respectively. We later mapped the genes using a backcross population derived from a cross between Swarna and RPBio4918-230S. We identified specific marker loci, namely RM8213, RM5953, and R4M17, on chromosome 4, flanking the *bph39(t)* and *bph40(t)* loci. Furthermore, quantitative expression analysis of candidate genes situated between the RM8213 and R4M17 markers was conducted. It was observed that eight genes exhibited up-regulation in RPBio4918-230S and down-regulation in Swarna after BPH infestation. One gene of particular interest, a serine/threonine-protein kinase receptor (*STPKR*), showed significant up-regulation in RPBio4918-230S. In-depth sequencing of the susceptible and resistant alleles of *STPKR* from Swarna and RPBio4918-230S, respectively, revealed numerous single nucleotide polymorphisms (SNPs) and insertion–deletion (InDel) mutations, both in the coding and regulatory regions of the gene. Notably, six of these mutations resulted in amino acid substitutions in the coding region of *STPKR* (R5K, I38L, S120N, T319A, T320S, and F348S) when compared to Swarna and the reference sequence of *Nipponbare*. Further validation of these mutations in a set of highly resistant and susceptible backcross inbred lines confirmed the candidacy of the *STPKR* gene with respect to BPH resistance controlled by *bph39(t)* and *bph40(t)*. Functional markers specific for *STPKR* have been developed and validated and can be used for accelerated transfer of the resistant locus to elite rice cultivars.

## 1. Introduction

Rice production is often hampered by the detrimental impact of insect pests, with the brown planthopper (BPH), *Nilaparvata lugens* (Stål) (Homoptera: Delphacidae), being a notorious phloem sap feeder responsible for causing yield losses of up to 60% [1,2,3]. BPH infestations result in an estimated annual economic loss exceeding USD 300 million in rice-producing regions of Asia [4,5]. In recent years, the southern part of India alone witnessed an annual paddy loss estimated at 64,602 quintals, amounting to USD 12,34,085 in financial terms due to BPH. The insistent damage caused by BPH, coupled with the adverse effects of pesticide applications, has impelled researchers to seek novel genetic resources conferring BPH resistance for integration into rice breeding programs.

Wild rice species serve as reservoirs for numerous valuable genes, particularly those associated with resistance to major biotic and abiotic stresses [6]. Among the 40 BPH resistance genes identified to date, 18 originate from wild rice species. These include *Bph11*, *bph12*, *Bph14*, *Bph15*, and *Bph16* from *Oryza officinalis*; *Bph13* from *Oryza eichingeri*; *Bph10* and *Bph18* from *Oryza australiensis*; *Bph20* and *Bph21* from *Oryza minuta*; *Bph22* from *Oryza glaberrima*; *Bph24*, *Bph27*, *bph29*, *Bph35*, and *Bph36* from *Oryza rufipogon* [7,8]; and *Bph34*, *bph39(t)*, and bph40(t) from *Oryza nivara* [9,10]. While many of these loci have been mapped, only a few have been functionally characterized, shedding light on gene expression patterns and comprehensive gene sequence characterization.

The BPH resistance present in rice cultivars with narrow genetic diversity has become susceptible to breakdown due to the emergence of new BPH biotypes. Thus, expanding the genetic base of rice cultivars is essential to combating this pest effectively. It is imperative to diversify the rice gene pool by identifying and incorporating new genes or quantitative trait loci (QTLs) for resistance from diverse sources, particularly wild rice relatives. Notably, most reported BPH-resistance genes are ineffective against BPH biotype 4, which is predominant in the Indian subcontinent. Furthermore, the majority of known BPH resistance genes have been identified primarily through seedling-stage screening, with limited information available on genes contributing to BPH resistance during the adult plant and reproductive stages. Therefore, it is crucial to identify BPH resistance genes against biotype 4 that manifest resistance phenotypes throughout various growth stages.

In a previous study, we harnessed the genetic resources of an introgression line RP4918-230S (IC0632075; INGR19041) developed from a wild rice species, *Oryza nivara* (accession IRGC 81848), which exhibited broad-spectrum resistance to BPH biotype 4 [10]. Inheritance analysis indicated that the BPH resistance followed a segregation ratio of resistant to susceptible plants in BC_1_F_2_ families, fitting into ratios of 7:9 and 1:3 through chi-square (χ^2^) tests. This suggested the presence of a pair of recessive genes governing BPH resistance. Subsequently, 200 BC_1_F_3_ plants derived from randomly selected BC_1_F_2_ plants were phenotyped using the Standard Seedbox Screening Test (SSST). Once again, the chi-square test confirmed the goodness of fit among the observed ratios (χ^2^ = 0.03–1.2, *p* < 0.9), providing evidence for the involvement of two genes controlling BPH resistance in the population. These two genes were designated as *bph39(t)* and *bph40(t).*

Building upon these findings, the present study aimed to fine-map the locus containing *bph39(t)* and *bph40(t)*, identify the potential candidate gene(s) associated with these two resistance genes, and undertake their comprehensive characterization.

## 2. Materials and Methods

### 2.1. Plant Materials and Mapping Population

An advanced backcross breeding approach was employed to create the mapping population. Swarna (a widely cultivated high-yielding indica variety) was used as the female parent (recurrent parent), while RPBio4918-230S (IC0632075; INGR19041) served as the male parent (donor parent). Due to certain weedy traits observed in the introgression line RPBio4918-230S (developed from the Swarna*2/*Oryza nivara* cross), such as awning, grain shattering, and weak tillers, additional backcrosses were conducted with Swarna, resulting in BC_1_F_1_ individuals. A total of 47 BC_1_F_1_ plants were self-pollinated to generate BC_1_F_2_ populations. These 47 BC_1_F_2_ families, each consisting of 50–200 plants, were screened for BPH resistance to understand the inheritance pattern based on 0–9 scale damage scores [10]. Subsequently, a set of 200 BC_1_F_3_ individual plants, randomly selected from the 47 BC_1_F_2_ populations, were both phenotyped three times during the seedling stage using the Standard Seedbox Screening Test (developed at IRRI, Philippines) [11] and genotyped to map the BPH resistance genes/QTLs introgressed from *Oryza nivara*. Additional phenotyping occurred twice at tillering and reproductive stages under field conditions at the Andhra Pradesh Rice Research Institute, Maruteru, India (coordinates: 16°37′32″ N 81°44′20″ E), which is a hotspot for BPH. The mean of the data was used for phenotype analysis (published [10]).

### 2.2. DNA Extraction, PCR, and Electrophoresis

Total DNA was extracted from fresh seedling-stage leaves using the CTAB method [12]. DNA quality was assessed with 1% agarose gel electrophoresis to visualize the integrity or fragmentation (intact/sheared) of the DNA samples. PCR amplification was conducted with a reaction mixture composed of template DNA (2 μL, 50–100 ng/µL), forward and reverse primers (2 mM each), dNTPs (1.25 mM), Taq DNA polymerase (1 U), and 1× PCR buffer (Tris with 1.5 mM MgCl_2_), with the total volume adjusted to 10 μL using sterile distilled water. The thermal profile included initial denaturation at 94 °C for 5 min, followed by 35 cycles of denaturation at 94 °C for 30 s, primer annealing for 30 s, extension at 72 °C for 1 min, and a final extension at 72 °C for 7 min. The amplified products were loaded onto a 3.5% agarose gel and visualized via ethidium bromide staining.

### 2.3. Marker Analysis

A total of 1027 markers (comprising SSRs and In-Dels) distributed evenly across the 12 rice chromosomes were employed for a polymorphic survey between the resistant and susceptible parents. The mapping population was screened with these polymorphic SSR and In-Del markers to identify their linkage to the BPH resistance trait.

### 2.4. Targeted Mapping of BPH Locus

Linkage analysis and map construction were conducted using QTL IciMapping [13]. Linkage groups were established via two-point analysis with a log-likelihood of odds (LOD) score of 3.0 and a maximum recombination level of 0.3. Map distances were converted into centiMorgans (cM) using the Kosambi function. The identified QTL regions were then screened to identify candidate genes within a target region spanning 15.5 cM (or the position of linked markers from 4.0 Mbp to 11.9 Mbp). Genes associated with QTL regions were retrieved from the RAP-DB database (https://rapdb.dna.affrc.go.jp/, accessed on 10 October 2021) (Table S2). Ten genes were selected for further analysis based on their annotated functions and potential roles in defence (Table 1).

### 2.5. Sample Preparation for Quantitative Real-Time PCR (qRT-PCR)

Seeds of both Swarna and RPBio4918-230S were sown in small pots (of 15 cm diameter), and two pots of each genotype were maintained under control conditions in a glasshouse. One set of pots of each genotype was placed in small cages (70 cm × 75 cm wooden cages having glass panels on one side and wire mesh on all other sides) for BPH infestation, while the other set was kept in the glasshouse at 30 ± 5 °C with 60 ± 10% relative humidity (RH) under natural light/dark conditions. Seedlings at the 2–3 leaf stage were infested with first-instar BPH nymphs at a rate of 6 to 8 nymphs per seedling. After four days, Swarna seedlings showed 60–80% drying, while RPBio4918-230S remained green. At this stage, leaf samples from control and BPH-infested plants of both Swarna and RPBio4918-230S were collected for RNA isolation. Three individual plants were sampled for each genotype (representing three biological replicates), and the leaf samples were frozen in liquid nitrogen and stored at −80 °C for RNA isolation.

### 2.6. Total RNA Isolation and cDNA Synthesis

Total RNA isolation was performed using the RNeasy Plant Mini Kit (Qiagen, Hilden, Germany) according to the manufacturer’s instructions. RNA quality and quantity were assessed using a spectrophotometer (Nano-Drop ND-1000, Thermo Scientific, Waltham, MA, USA) and 1% agarose gel electrophoresis. RNA samples were treated with DNaseI and normalized for cDNA synthesis. For cDNA synthesis, 100 µg of RNA was used, employing the Improm-II Reverse Transcription System (Promega, Madison, WI, USA) as per the manufacturer’s instructions. cDNA was treated with RNAase for subsequent qRT-PCR analysis.

### 2.7. Quantitative Real-Time PCR (qRT-PCR)

Information about the 10 selected genes (Table 1) was retrieved from the RAP-DB, and primers for real-time PCR were designed (Table 2) using Primer 3.0 software (https://bioinfo.ut.ee/primer3-0.4.0/, accessed on 20 November 2021). qRT-PCR was conducted using SYBR Premix Ex-Taq (Takara, Tokyo, Japan) following the manufacturer’s instructions in a Light Cycler^®^ 96 Real-time PCR system (Roche, Basel, Switzerland). The rice ubiquitin gene served as the endogenous control. The qRT-PCR profile included a pre-holding stage at 50 °C for 10 min, a holding stage at 95 °C for 10 min, denaturation at 95 °C for 15 s, annealing/extension at 60 °C for 30 s for 40 cycles, followed by a disassociation stage (melting curve analysis) [14]. To determine relative expression levels, three independent biological replicates were used. Gene expression fold changes were calculated using the comparative threshold cycle (Ct) method, with ΔCT values normalized against the Ct value of the endogenous control. Further, ΔΔCT values were calculated as ΔCT of the infested sample minus ΔCT of the control sample, and gene expression fold differences were derived from 2^−ΔΔCt^. Additionally, the ΔCT standard deviation was computed as previously reported [15,16].

**Table 1:** Description of the ten genes selected for the expression study.

Gene ID	Gene Name	Gene Description
Os04g12540.1	*OsRLK-1*	receptor-like protein kinase, putative, expressed
Os04g12560.1	*OsRLK-2*	receptor-like protein kinase, putative, expressed
Os04g12580.1	*OsRLK-3*	receptor-like protein kinase, putative, expressed
Os04g15580.1	*STPKR*	serine/threonine-protein kinase receptor precursor, putative, expressed
Os04g15630.1	*Xa21*	xa21, putative, expressed
Os04g15650.1	*LRR*	leucine-rich repeat family protein, expressed
Os04g15660.1	*RK*	receptor kinase, putative, expressed
Os04g18650.1	*AP2/ERF*	APETALA2/ethylene-responsive element binding protein 34
Os04g19800.1	*F.BOX*	F-box domain containing protein, expressed
Os04g20180.1	*LGDSL*	lipase, GDSL-domain-containing protein

### 2.8. Sequencing and Data Analysis

To sequence the full-length serine/threonine-protein kinase receptor (STPKR) gene, including regulatory regions, five sets of overlapping primers (Table S3) were designed. PCR reactions used template DNA from both RPBio4918-230S (resistant) and Swarna (susceptible) and followed an initial denaturation at 98 °C for 5 min, 35 cycles of denaturation at 98 °C for 30 s, primer annealing at 60 °C for 30 s, extension at 72 °C for 1 min, and a final extension at 72 °C for 20 min. PCR amplicons were purified using the GeneJET purification kit (Thermo Scientific, Waltham, MA, USA) and sequenced at a commercial facility. A minimum of three PCR products from each primer set were sequenced. Sequenced fragments were aligned using BioEdit Sequence Alignment Editor Software ver. 7.2 (https://bioedit.software.informer.com/7.2/, accessed on 20 November 2021). The corresponding DNA sequences of Nipponbare (Os-Nipponbare-IRGSP-1.0) were used as a reference. Multiple alignments of DNA and protein sequences were performed using the BioEdit Sequence Alignment Editor Software. To identify genotype-specific cis-acting regulatory motifs and associated variations between RPBio4918-230S and Swarna, a Plant Cis-acting Regulatory DNA Elements tool called New PLACE was employed (https://www.dna.affrc.go.jp/PLACE/?action=newplace, accessed on 20 November 2021).

## 3. Results

### 3.1. Construction of Linkage Map and Mapping of BPH-Resistant QTLs

In our investigation, we initially assessed 25 markers associated with known BPH-resistant genes implicated in defence against BPH biotype 4. The outcomes revealed the absence of the resistant allele related to these known genes in *Oryza nivara* [10]. Subsequently, we conducted a comprehensive survey using a total of 991 SSR and 36 In-Del markers evenly distributed across all 12 rice chromosomes to identify polymorphic markers. Thirteen polymorphic markers were identified on chromosomes 1, 4, and 10, differentiating between the resistant (RPBio 4918-230S) and susceptible (Swarna) parental lines.

**Figure 1:**
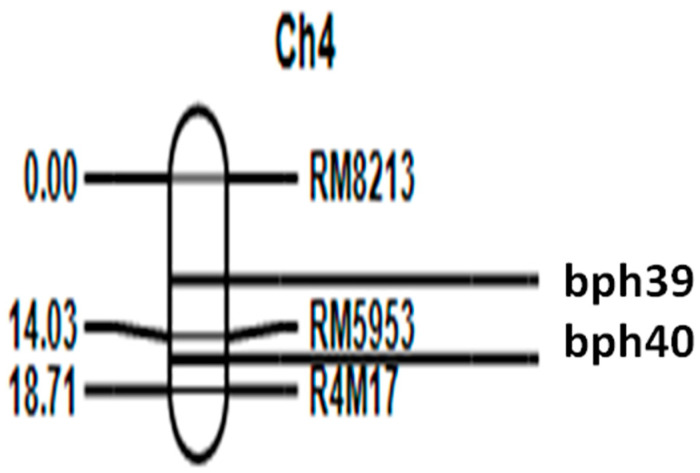
Illustration of the molecular linkage map of SSR markers associated with the loci responsible for BPH resistance, along with their respective positions, generated using IciMapping V4.1.

**Figure 2:**
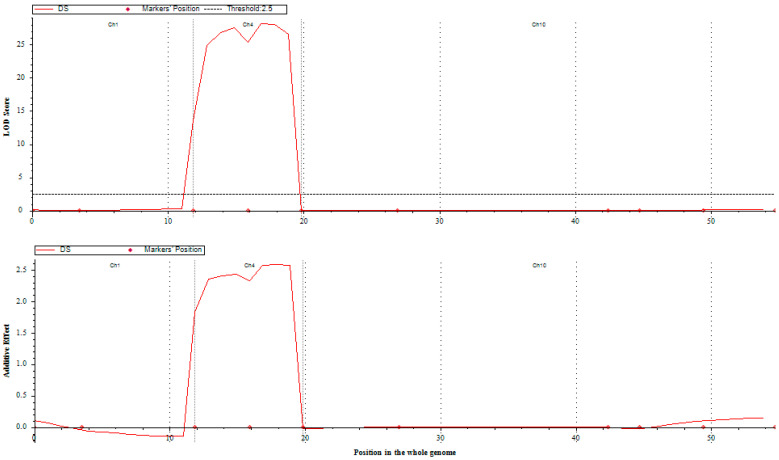
Presentation of the QTL likelihood curve depicting the LOD score for BPH damage score (DS). These QTLs were identified on chromosomes using QTL IciMapping version 4.1. The graph displays the LOD score curve along with the additive effects of the identified QTLs. DS = damage score.

A set of 200 BC_1_F_3_ lines were both phenotyped and genotyped. Phenotyping involved rigorous screening at the seedling and tillering stages. Simple interval mapping unveiled two major loci, *bph39* and *bph40*, on chromosome 4. The *bph39* locus was mapped between markers RM8213 (physical distance of 4.4 Mbp) and RM5953 (physical distance 9.4 of Mbp), with an LOD value of 26.5 at the peak position and a distance of 3.0 cM. Similarly, the *bph40* locus was identified in the region between RM5953 and R4M17 (physical distance 11.4 of Mbp), with an LOD value of 27.0 at the peak position and a distance of 6.0 cM. Specifically, bph39 was positioned 1.5 cM to the left of RM8213 and 3.5 cM to the right of RM5953, while bph40 was situated 4.5 cM to the left of RM5953 and 7.0 cM to the right of R4M17 (Table S1 and Figure 1). These two loci, *bph39* and *bph40*, accounted for 21% and 22% of the total phenotypic variance, respectively. Importantly, the additive effect of the damage score was positive (Figure 2), suggesting that the BPH resistance alleles were contributed by the male donor parent, RPBio4918-230S.

### 3.2. Identification and Expression Analysis of BPH-Resistant Candidate Genes

The genomic regions associated with BPH resistance genes were found to share common stretches within the 4.4 to 9.6 Mb region of chromosome 4. Analysis of these mapped regions revealed that approximately 18% of the genes within these loci were associated with defence mechanisms, while nearly 45% fell into categories related to protein degradation, modification, sorting, chaperones, redox processes, and defence proteins [17].

**Figure 3:**
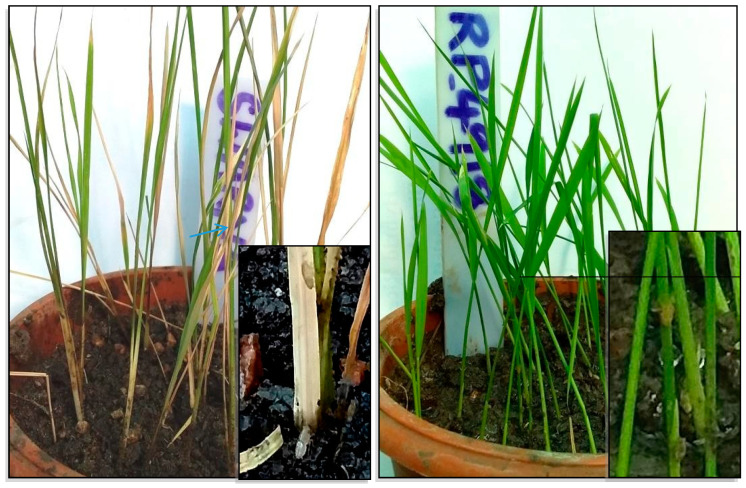
Depiction of the condition of plants after 3 days of BPH infestation. Notably, the nymphs on the susceptible parent, Swarna, have progressed to the third and fourth instar stages. In contrast, the nymphs on the resistant parent remain in the second instar stage due to inadequate feeding.

From the pool of genes present within the mapped loci (Table S2), we selected 10 candidates for further analysis based on their annotated functions, targeting them for qRT-PCR investigation (Table 1). Utilizing the reference genomic sequence (Os-Nipponbare-IRGSP-1.0), we designed gene-specific primers (Table 2). To evaluate the expression of these genes, we subjected 16-day-old plants to BPH infestation, which resulted in noticeable differences in damage levels and nymph growth. Nymphs on the susceptible parent, Swarna, progressed to the third and fourth instar stages, while nymphs on the resistant parent remained in the second instar stage due to inadequate feeding (Figure 3).

**Figure 4:**
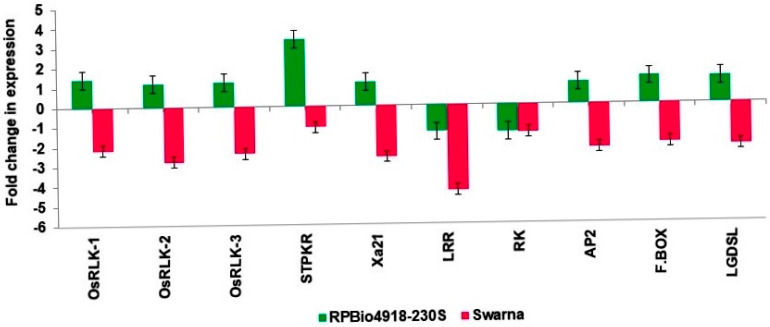
Illustration of the differential expression patterns of genes as determined with qRT-PCR. It depicts the up-regulated and down-regulated genes among the commonly identified differentially expressed genes (DEGs) in RPbio4918-230S (RP) and Swarna (SW). Notably, there is a higher prevalence of up-regulated DEGs in RPbio4918-230S among the common set.

Among the 10 selected candidate genes, 8 displayed distinct expression patterns between RPBio4918-230S and Swarna in response to BPH infestation. Compared to the control, genes such as *OsRLK-1*, *OsRLK-2*, *OsRLK-3*, *STPKR*, *Xa21*, *AP2*, *F.BOX*, and *LGDSL* exhibited up-regulation in RPBio4918-230S and down-regulation in Swarna after BPH infestation. Conversely, *LRR* and *RK* showed down-regulation in both genotypes following BPH infestation. Notably, the most significant up-regulation, reaching a fold change of 3.3-fold, was observed in the serine/threonine-protein kinase receptor (*STPKR*) gene expression in the resistant parent, RPBio4918-230S, while it was down-regulated in the susceptible parent, Swarna (Figure 4).

### 3.3. Sequence Variation Analysis of the STPKR Gene

To compare the R allele (RPBio4918-230S) with the S allele (Swarna) of the STPKR gene, we designed five sets of overlapping primers based on the reference *STPKR* gene sequence (Os-Nipponbare-IRGSP-1.0). The reference STPKR gene, with the locus name LOC_Os04g15580.1, was situated on chromosome 4 within the region spanning 8472824-8476142. The retrieved *STPKR* gene had a length of 3319 base pairs and included distinct regions such as a 5’ untranslated region (UTR) of 1177 base pairs, 7 exons, 6 introns, and a 3’ UTR of 110 base pairs. The gene’s seven exons encoded a protein consisting of 478 amino acids with an estimated molecular weight of approximately 53.0 kDa.

We employed the designed gene-specific primers to amplify the full-length *STPKR* gene, encompassing regulatory sequences. When comparing RPBio4918-230S to Swarna, we observed differences in the form of 15 transversions and 8 transitions within the exonic regions (Table 3. Specifically, among the seven exons, exons 1 and 2 exhibited four single nucleotide polymorphisms (SNPs) each, exon 3 had one SNP, exon 4 had no SNPs, exon 5 had two SNPs, exon 6 showed seven SNPs, and exon 7 contained five SNPs between RPBio4918-230S and Swarna. Moreover, a significant number of SNPs were identified within the intronic regions of both genotypes, suggesting their potential role in gene expression and regulation through genetic and epigenetic mechanisms (Figure S1).

### 3.4. Translated Peptide Sequences and Amino Acid Variations

**Figure 5:**
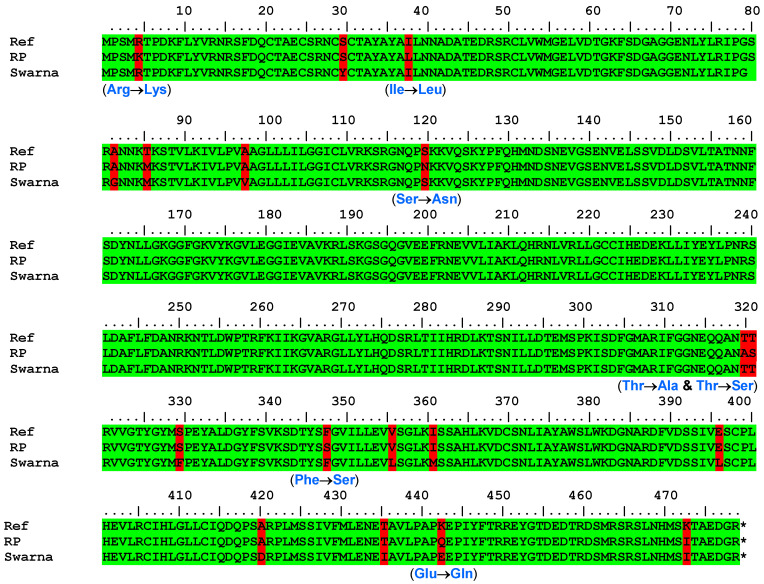
Protein sequence alignment of STPKR gene for RPbio4918-230(S) and Swarna.

Both parental lines, RPBio4918-230S and Swarna, exhibited translated peptide sequences composed of 478 amino acids, mirroring the reference sequence of *Nipponbare*. Upon comparing the amino acid sequences between RPBio4918-230S and Swarna, we identified 16 differences, namely R5K, Y30S, I38L, G82A, V98A, S120N, T319A, T320S, F330S, F348S, L356V, M361I, L396E, D420A, I435T, and E443Q (see Figure 5). Most of these mutations were located in the N and C terminal regions of the protein, with the central domain displaying a high degree of conservation. Among the identified mutations, R5K, I38L, S120N, T319A, T320S, and F348S were particularly noteworthy, as they represented distinctive amino acid substitutions in RPBio4918-230S when compared to Swarna and Nipponbare.

To further validate the stability of these promising mutations at the exonic regions, we conducted resequencing of the *STPKR* gene in a set of five BPH-resistant and susceptible backcross inbred lines (BILs). The *STPKR* gene was isolated and sequenced from each individual line. As anticipated, the sequence reads from both resistant and susceptible individual lines exhibited complete homology with RPBio4918-230S and Swarna, respectively. The mutations R5K, I38L, S120N, T319A, T320S, and F348S were clearly observed in the sequences of the contrasting BILs.

### 3.5. Variations in Regulatory Regions of STPKR Gene

In the examination of variations within the regulatory regions of the STPKR gene between RPBio4918-230S and Swarna, a total of 46 single nucleotide polymorphisms (SNPs) were identified. Out of these, 45 were located in the 5’ untranslated region (5’UTR) and 1 in the 3’ untranslated region (3’UTR). Intriguingly, among the 45 SNPs within the 5’UTR, 22 were situated in RPBio4918-230S, while 24 were found in Swarna, and these SNPs were positioned in critical cis-acting regulatory elements with the potential to influence gene transcription.

In RPBio4918-230S, 33 functional cis-regulatory domains were identified (31 within the 5’UTR and 2 within the 3’UTR region), all of which exhibited sequence variations compared to Swarna at the respective motifs (see Table S4). Similarly, in Swarna, 43 functional cis-regulatory domains were identified (41 within the 5’UTR and 2 within the 3’UTR region), with corresponding motif variations observed in comparison to RPBio4918-230S (see Table S5).

## 4. Discussion

The majority of the BPH resistance genes identified in *Oryza sativa* and other wild species of rice are reported to show resistance reactions to BPH biotypes 1, 2, or 3 but are ineffective against BPH biotype 4, which is prevalent in the Indian subcontinent and thus noted as the most destructive biotype [9,10]. Considering the evolution of BPH biotypes and the ineffectiveness or breakdown of genetic resistance in rice genotypes, a catalogue of new genes needs to be identified for each biotype to ensure the durability of host plant resistance. In this study, we have mapped a novel BPH resistance genetic locus in the introgression line RPBio4918-230S developed from *Oryza nivara* that confers resistance to BPH biotype 4. The markers used for the polymorphic survey covered the whole genome, ensuring that all the introgressed segments could be mapped. Theoretically, the average alien chromatin content in BC_1_Fn generation is expected to be about 25%, or we can say that after BC_1_Fn, there is a probability of 75% of Swarna background recovery with only 25% of the donor genome. Since the donor RPBio4918-230S was developed after two backcrosses of Swarna, the lines were selected in the BC_2_F_6_ generation after selfing and selection in each generation, which resulted in a significant recovery of the Swarna genome. To further develop the mapping population, RPBio4918-230S was again crossed and backcrossed with Swarna. This resulted in a total of four backcrosses with the maximum genome recovery of Swarna, for which only a few markers could show polymorphism.

As the RPBio4918-230S-derived mapping population (BC_1_F_3_) is developed in the background of Swarna, a considerable selection differential against alien chromatin (*Oryza nivara*) was observed. Hence, with a maximum background of Swarna out of the total 1027 markers surveyed, only 13 polymorphic markers were identified, indicating 1.3% of the *Oryza nivara* genome in the progenies. Introgression segments retained on chromosomes 1, 4, and 10 were probably those that became free of linked negative alleles during early recombination events or had a net positive effect on the BPH-resistant trait. Cheema et al. [18], while working with the *Oryza rufipogon*/IR64 BC_2_F_6_ population, also obtained a very low introgression rate of 7% with polymorphisms on chromosomes 1, 2, 3, 5, 6, and 11 only. Miah et al. [19] also reported the recurrent parent genome content in the selected BC_2_F_2_ lines ranging from 92.7% to 97.7% using SSR markers. Chen et al. [20], in their study of genetic diversity and phenotypic variation in an introgression line population of an interspecific *Oryza glaberrima* and *Oryza sativa* (RAM3/Jin23B//Jin23B///YuetaiB), revealed the proportion of the *Oryza glaberrima* genome (PGG) in the ILs ranging from 0.3% to 36.7%, with an average value of 12.32%.

The BC_1_F_3_ population, derived through an advanced backcross breeding strategy, was used to construct the genetic map of two BPH resistance loci, *bph39* and *bph40*, located on chromosome 4 in the genomic region closely linked to RM8213, RM5953, and R4M17. Previously, eight other BPH-resistance genes have been reported on this chromosome 4, viz., *Bph6* [21], *Bph12* [22], *Bph15* [23], *Bph17* [24], *Bph20(t*) [25], *Bph27* [26], *Bph33* [27], *Bph33(t*) [28], and *Bph34* [9]. Among these, six genes were identified in different wild species. *Bph 6* showed resistance to only the Bangladesh biotype, *Bph12* and *Bph15* showed resistance to biotypes 1 and 2, *Bph20* showed resistance to biotype 1 only, and *Bph 27* showed resistance to biotype 2 and was predominant in China [26].

Of the BPH resistance genes mapped on chromosome 4, *Bph17*, discovered from the Sri Lankan landrace Rathu Heenati, is found nearer to the *bph39* locus, which was mapped in this study. *Bph17* was reported at map distances of 3.2 cM from RM5953, while the mapped QTL in this study is 3.5 cM from RM5953. Furthermore, resistance screening showed a differential response to resistance, as RPBio4918-230S was found to be resistant at the maximum tillering stage while Rathu Heenati did not show tolerance at this stage [29]. It has also been reported that Rathu Heenati is resistant to all four BPH biotypes at the seedling stage but susceptible at the tillering stage [30]. In order to support that different genes or alleles in this locus might be governing the BPH resistance phenotype at seedling (in Rathu Heenati) and seedling/tillering (in RP bio 4918-230S) growth stages, the allelism test was conducted in our previous study, which indicated that Rathu Heenati and RPBio4918-230S amplified different alleles with markers RM8213 and RM5953 linked to *Bph17* [10]. The cloning of candidate genes from the *Bph17* locus revealed that it is a cluster of three genes (*OsRLK-1*, *OsRLK-2*, and *OsRLK-3*) governing the BPH resistance trait. Notably, presence of another lectin receptor kinase gene located immediately outside the 79 kb cloned gene cluster of *Bph17* was also suggested. A sequence comparison of these genomic sequences showed maximum similarity between Rathu Heenati and *Oryza nivara* [31]. In another study, Rathu Heenati and *Oryza. nivara* were grouped in a major cluster while studying the genetic differentiation among Sri Lankan traditional rice varieties and wild rice species using AFLP markers [32]. Taken together, these studies and our findings suggest that *Bph17* might have originally evolved from *Oryza nivara*, the progenitor of cultivated rice in Asia and an important donor of BPH resistance, and later introgressed into other genotypes, including Rathu Heenati, from any of the parents in their genealogy having *Oryza nivara* chromosome segments. However, in this process of evolution, different alleles might have evolved, giving differential resistance to BPH at the seedling and tillering stages. It would be interesting to further probe this genetic locus in *Oryza nivara* accessions to better understand the mechanisms of BPH resistance in rice. Sarao et al. [33] studied around thousands of accessions of wild rices and found *Oryza nivara* as the potential source of not only BPH resistance but also for disease (grassy stunt, bacterial blight, and sheath blight) resistance and productivity-related traits. In addition to *bph39*, we identified another major locus, *bph40*, contributing to BPH resistance. Further dissection of these two novel loci will help in identifying the genes and mechanisms associated with resistance against BPH biotype 4 at the seedling and tillering growth stages of rice.

The expression analysis of three genes (*AP2/ERF*, *F.BOX*, and *GDSL*) located in the *bph40* locus showed up-regulation in RPBio4918-230S and down-regulation in Swarna after the BPH infestation. The up-regulation of the Os04g18650.1 encoding the *AP2/ERF* gene present in this mapped locus of resistant parents indicates the important role of the ethylene pathway in the BPH resistance phenotype. In an interesting study, Hu et al. [34] showed that BPH feeding rapidly initiated the ethylene signalling pathway. While examining the resistance mechanism of the brown-planthopper-induced 008a (Bphi008a; AY256682) gene, overexpression of Bphi008a enhances the expression of *AP2/ERF* transcription factors. It is noteworthy that *AP2/ERF* transcription factors play an important role in ethylene and jasmonate-pathway-mediated plant defence [35]. Similarly, induced expression of Os04g20180.1 encoding lipase and the *GDSL*-domain-containing protein in RPBio4918-230S indicates the important role of lipids and lipid metabolites in BPH resistance. *GDSL* lipases play an important role in the immunity response in rice through lipid homeostasis [36]. A strong increase in *GDSL* lipase expression was shown in the proteome profile of *Plutella xylostella* larvae-infested leaves of *Arabidopsis thaliana* [37]. Deciphering the role of *GDSL* lipases in BPH resistance will further broaden the understanding of the resistance mechanisms of rice against this sucking pest.

Similarly, expression analysis of candidate genes underlying the *bph39* locus showed the up-regulation of *OsRLK-1*, *OsRLK-2*, *OsRLK-3*, *STPKR*, and *Xa21* in resistant parent RPbio4918-230S, while these genes were down-regulated in susceptible parent Swarna after the BPH infestation. Among these, three candidate genes (*OsRLK-1*, *OsRLK-2*, and *OsRLK-3*) reported in the resistance gene cluster of *bph*17 have been characterized [31]. Further, Liu et al. [31] discussed the possibility that some other genes located in this region could contribute to BPH resistance. The chromosomal segment transferred/introgressed in RPBio4918-230S is conferring more robust resistance than Rathu Heenati, specifically at the tillering stage, indicating the introgression of additional genes in RPBio4918-230S from the original progenitor, *Oryza nivara*. Induced expression of *Os04g15630.1* encoding *Xa21* (an uncharacterized protein) supports the possibility of more genes in this locus contributing to the BPH resistance trait, which needs to be delineated and analyzed in further studies [38]. In this study, we noticed the maximum up-regulation of a serine/threonine-protein kinase receptor precursor (*STPKR*) in RPBio4918-230S after BPH infestation, which was even higher than previously reported BPH resistance genes *OsRLK-1*, *OsRLK-2*, and *OsRLK-3* present in this locus. The important role of the serine/threonine-protein kinase receptor in signalling and plant defence has been reported in various studies [39,40]. Lin et al. [41] reported a serine/threonine-protein-kinase-mediated regulation of immune responses in *Arabidopsis*. 

The maximum expression of the *STPKR* gene in RPBio4918-230S under BPH infestation motivated us to further probe the structural genomics of susceptible and resistant alleles. The complete gene sequencing, including its regulatory regions from Swarna and RPBio4918-230S, helped with the identification of SNPs/In-DELs that might have a significant role in resistance response. A comparison of two genotypes revealed 15 transversions and 8 transitions in the coding region and 46 SNPs in the regulatory sequences of the *STPKR* gene, which suggested that the S and R alleles are structurally different. Also, a number of intronic SNPs were noticed that might play a crucial role in gene expression and regulation through genetic and epigenetic pathways [16]. Among all the mutations, R5K, I38L, S120N, T319A, T320S, and F348S seem to be the most promising, as they were distinct in RPBio4918-230S when compared with Swarna and the reference sequence of *Nipponbare*. Further, these mutations were verified by sequencing the corresponding nucleotide regions in a set of highly susceptible and resistant BILs. All the susceptible BILs showed Swarna-type sequences, while the resistant BILs showed RPBio4918-230S-type sequences. The structural analysis of the *STPKR* gene indicates it is the most potential target for further characterization through over-expression/silencing approaches. Also, the deeper analysis of identified SNPs corresponding to R5K, I38L, S120N, T319A, T320S, and F348S may help narrow down the causal SNP contributing to the resistant phenotype. This can be utilized later to create resistant alleles in BPH susceptible high-yielding genotypes through base editing or genome editing. 

## 5. Conclusions

This study has successfully mapped novel loci, *bph39* and *bph40*, which harbour BPH resistance genes derived from the wild rice species *Oryza nivara*. These loci have demonstrated their effectiveness in conferring resistance against BPH biotype 4 at various growth stages, including the seedling and tillering stages. Additionally, our investigation into candidate genes within these mapped loci has revealed distinct expression patterns in both resistant and susceptible parents following BPH infestation.

Furthermore, a comprehensive examination of the serine/threonine-protein kinase receptor precursor (*STPKR*) gene at the structural genomics level has unveiled potential SNPs and mutations that play a pivotal role in the BPH resistance phenotype. The future cloning and functional characterization of these identified genes promise to enhance our comprehension of the mechanisms underpinning BPH resistance in rice. This knowledge can, in turn, be harnessed to breed rice cultivars endowed with robust, broad-spectrum resistance against various BPH biotypes. Additionally, the identification of causal SNPs and genetic markers will facilitate marker-assisted breeding and genome editing in popular rice cultivars, ushering in a new era of pest-resistant rice varieties.

## Figures and Tables

**Table 2:** List of primers designed for real-time PCR using Primer 3.0 software.

Gene Name	Forward Primer and Reverse Primers	Size
*OsRLK-1*	GACCCAAGCATGCGACCTAA	
	AGAAGCGTATGGAAGTGAGC	114 bp
*OsRLK-2*	GCTCCTCTTTGACACCCCAG	
	CAGCGAGCAGGTATGAGGAG	111 bp
*OsRLK-3*	GGCTGTGTGTGACTGATTGT	
	TGTCCTCGGTACATTGACATCC	119 bp
*STPKR*	TGGCTCACGAGCTAATAACAA	
	TGATTGCCTCTTGATTTGCGTA	120 bp
*Xa21*	CGCGCGTCTAGGTGATTTTG	
	CATTCCATACTCTGGTGCAATGT	115 bp
*LRR*	GCGCTTCTTGGTGACTTTGG	
	CCCATTCCATACTCTGGTGCAAT	116 bp
*RK*	GTACCAAGGCAGCCCAAAGA	
	CACAGTGGATCAGAGGAGGT	119 bp
*AP2/ERF*	GAGGACTACAGCTACCGCAA	
	TAGCAATCCGGGTCTCGTTT	115 bp
*F.BOX*	GGAAACGTCTCGGGACAGAA	
	GGTCGTAAGGCGGGTAGAAC	115 bp
*LGDSL*	CGGGATCACTGCCAGAAAAA	
	GCACGGATTTGGAAGGGCT	119 bp

**Table 3:** Sequence differences in coding region of serine/threonine-protein kinase receptor gene of RP bio4918-230S in comparison to Swarna.

S. No	Nucleotide Change in RP bio4918-230S	Position of the Nucleotide Change	Type of Change	Exon Number
1	G→A	994 bp	Transition	1
2	A→C	1069 bp	Transversion	1
3	A→C	1092 bp	Transversion	1
4	C→T	1103 bp	Transition	1
5	G→C	1359 bp	Transversion	2
6	G→A	1384 bp	Transition	2
7	T→C	1407 bp	Transition	2
8	A→T	1429 bp	Transversion	2
9	G→A	1561 bp	Transition	3
10	A→G	2350 bp	Transition	5
11	A→T	2353 bp	Transversion	5
12	T→C	2480 bp	Transition	6
13	C→G	2512 bp	Transversion	6
14	T→C	2535 bp	Transition	6
15	T→A	2539 bp	Transversion	6
16	T→G	2558 bp	Transversion	6
17	G→C	2575 bp	Transversion	6
18	C→A	2617 bp	Transversion	6
19	T→G	2762 bp	Transversion	7
20	T→A	2763 bp	Transversion	7
21	A→C	2835 bp	Transversion	7
22	T→C	2880 bp	Transition	7
23	G→C	2900 bp	Transversion	7

## Data Availability

All data supporting the findings of this study are available within the paper and its Supplementary Information.

## Supplementary Materials

The following supporting information can be downloaded at https://www.mdpi.com/article/10.3390/genes14112066/s1. Figure S1: Multiple sequence alignment of nucleotide sequences of serine/threonine-protein kinase receptor (STPKR) gene Table S1: Summarization of the QTLs that were identified in the backcross introgression mapping population of Swarna × RPbio4918-230S for BPH resistance, employing QTL IciMapping V 4.1., Table S2: List of genes in the mapped region Table S3: Five overlapping primers designed to cover the whole candidate gene. Table S4: In silico analysis for the variations at the Cis-regulatory motifs in the form of SNPs at the promoter/regulatory regions of the differentially expressed OsSTPKR gene in RPbio4918-230(S) vs. SWARNA Table S5 In silico analysis for the variations at the Cis-regulatory motifs in the form of SNPs at the promoter/regulatory regions of the differentially expressed OsSTPKR gene in SWARNA vs. RPbio4918-230(S). References [42,43,44,45,46,47,48,49,50,51,52,53,54,55,56,57,58,59,60,61,62,63,64,65,66,67,68,69,70,71,72,73,74,75,76,77,78,79,80,81,82,83,84,85,86,87] are cited in the Supplementary Materials.
