# Supplementary material for: Identification and Functional Characterization of Two Major Loci Associated with Resistance against Brown Planthoppers (Nilaparvata lugens (Stål)) Derived from Oryza nivara
Source: Genes (Basel). 2023 Nov 11;14(11):2066. doi: 10.3390/genes14112066 (PMC10671472; doi:10.3390/genes14112066)
Supplement: Supplementary file 1 [file genes-14-02066-s001.zip › Figure S1.pdf]

Supplementary Figure S1. Multiple sequence alignment of nucleotide sequences of serine/threonine-protein kinase receptor (STPKR) gene. The sequences were generated for RPbio4918-230(S) and Swarna and compared with reference sequence (Os-Nipponbare-IRGSP-1.0) available in RAP-DB database. Different elements of genes are shaded with different colours (described at the end of the figure), while mutations (SNPs/In-Dels) are shown without colour shade.

```
                  10        20        30        40        50        60        70        80
          ....|....|....|....|....|....|....|....|....|....|....|....|....|....|....|....|
Ref       TCATCTCCAGTGGCGGCGTCTTCGCTCTCGGCTTCTTCTCCCTGACGAACTCAACCTCGGACTTGTACGTCGGCGTATGG
Swarna    TCATCTCCAGCGGCGGCGTCTTCGCTCTCGGCTTCTTCTCCCCGACGAGCTCAACTTCGGACTTGTACGTCGGCGTGTGG
RP        TCATCTCCAGCGGCGGCGTCTTTGCTCTCGGCTTCTTCTCCCCAACGAGCTCAACTTCGGACTTGTACGTCGGCGTGTGG

                  90       100       110       120       130       140       150       160
          ....|....|....|....|....|....|....|....|....|....|....|....|....|....|....|....|
Ref       TACAACCAGATCCCCGTGCACACCTACGTGTGGGTCGCCAACCGCAACACCCCGATCAAGAAGAGCTCCTCCGTGAAGCT
Swarna    TACAACCAGATCCCCGTGCGCACCTACGTGTGGGTCGCCAACCGCAACACCCCGATCAAGAAGAGCTCCTCCGTGAAGCT
RP        TACAACCAGATCCCCGTGCGCACCTACGTGTGGGTCGCCAACCGCAACACCCCGATCAAGAAGAGCTCCTCCGTGAAGCT

                 170       180       190       200       210       220       230       240
          ....|....|....|....|....|....|....|....|....|....|....|....|....|....|....|....|
Ref       GGTCTTGACCAACGATTCCGACCTCGTGCTGTCCGACTCCAATGGTGGCGGCGGCGGCGGCGCCGTCTGGACGACGGCGA
Swarna    GGTCTTGACCAACGATTCCGACCTCGTGCTGTCCGACTCCAATGG---CGGCGGTGGCGGCGCCGTCTGGACGACGGTGA
RP        GGTCTTGACCAACGATTCCGACCTCGTGCTGTCCGACTCCAATGG---CGGCGGTGGCGGCGCCGTCTGGACGACGGTGA

                 250       260       270       280       290       300       310       320
          ....|....|....|....|....|....|....|....|....|....|....|....|....|....|....|....|
Ref       ATTCGAACAACGTCGCCGCCGCCGGTGGTGGTGCTGGAGCGACGGCTGTGCTGCTCGACTCCGGCAACTTCGTCGTGCGG
Swarna    A------CAAAGTCGCCGCCGCCGGTGTTGGTGCTGGAGCAACGGCTGTGCTGCTCGACTCCGGCAACTTCGTTGTGCGG
RP        A------CAACGTCACCGCCGCCGGTGTTGGTGCTGGAGCAACGGCTGTGCTGCTCGACTCCGGCAACTTCGTTGTGCGG

                 330       340       350       360       370       380       390       400
          ....|....|....|....|....|....|....|....|....|....|....|....|....|....|....|....|
Ref       CTGCCGAACGGGTCTGAGGTATGGCGGAGCTTCGATCACCCGACGGACACCATCGTGCCCAACGTGAGTTTCTCGCTGAG
Swarna    CTGCCGAACGGCTCGGAAGTATGGCGGAGCTTCGATCACCCGACGGACACCATCGTGCCCAACGTGAGTTTCCCGCTGAG
RP        CTGCCGAACGGCTCTGAAGTATGGCGGAGCTTCGATCACCCGACGGACACCATCGTGCCCAACGTAAGTTTCTCGCTGAG

                 410       420       430       440       450       460       470       480
          ....|....|....|....|....|....|....|....|....|....|....|....|....|....|....|....|
Ref       CTACATGGCCAACAGCTTAGACCGCATCGTCGCGTGGAGGGGGCCCAACGACCCGTCCGCCGGCGACTTCACCATGGGCG
Swarna    CTACATGGCCAACAGCTTAGACCGCATCGTCGCGTGGAGGGGGCCCAACGACCCGTCCGCCGGCGACTTCACCATGGGCG
RP        CTACATGGCCAACAGCTTATACCGCATCGTCGCGTGGAGGGGGCCCAACGACCCGTCCTCCGGCGAGTTCGCCATGGGCG

                 490       500       510       520       530       540       550       560
          ....|....|....|....|....|....|....|....|....|....|....|....|....|....|....|....|
Ref       GCGACT------------------CCAGCTCGGACCTCCAGATCGTCGTCTGGAACGG--GACGAGGCCGTACTGGCGAC
Swarna    GCGACTTCACCATGGGCGGCGACTCCAGCTCGGACCTCCAGATCGTCCTCTGGAACGG--GACGAGGCCGTACTGGCGAC
RP        GCGACT------------------CCATCT-GGACGTCCAGATCGTCTTCTGGAATCGTGGAAGAGGCCGTACTGGCGAC

                 570       580       590       600       610       620       630       640
          ....|....|....|....|....|....|....|....|....|....|....|....|....|....|....|....|
Ref       GAGCGGCGTGGACCGGCGCGTCCATCTTCGGCGTGATCCAGACCAACACCAGCTTCAAGCTGTATCAGACGATCGACGGC
Swarna    GAGCGGCGTGGACCGGCGCGTCCATCTTCGGCGTGATCCAGACCAACACCAGCTTCAAGCTGTACCAGACGATCGACGGC
RP        GAGCGGCGTGGACCGGGGCGTCCATCTTCGTCGTGATCCAGATCAACACCAGCTTCAAGCTGTACCAGACGATCGACGGC

                 650       660       670       680       690       700       710       720
          ....|....|....|....|....|....|....|....|....|....|....|....|....|....|....|....|
Ref       GACATGGCCGACGGCTACTCCTTCAAGCTGACCGTCGCCGACGGCTCGCCGCCGATGCGCATGACGCTGGACTACACCGG
Swarna    GACTTGGCCGACGGCTACTCCTTCAGGCTGACCGTCACCGACGGCTCGCCGCCCATGCGCATGACGCTGGACTACACTGG
RP        GACATGGCCGACGGCTACTCCTTCAAGCTGACCGTCGCCGTCGGCTCGCCGTCGATGCGCATGACGCTCGACTACACTGG

                 730       740       750       760       770       780       790       800
          ....|....|....|....|....|....|....|....|....|....|....|....|....|....|....|....|
Ref    CGAGCTCACCTTCCAGAGCTGGGACGGCAACACGTCGTCGTGGACCGTCTTCTCCCGGTTCCCCACCGGCTGTGACAAG
```

```
Swarna      CGAGCACACCTTCCAGAGCTGGGACGGCAACACGTCGTCGTGGACCGTCTTCGCCCGGTACCCCATCGGCTGCGACAAGT
RP          CGACCACACCTTCCAGAGCTGGGACGGCAACACGTCATCGTGGACCGTCTTCGCCCGGTTCCCCATCGGCTGCGACAAGT

                     810       820       830       840       850       860       870       880
            ....|....|....|....|....|....|....|....|....|....|....|....|....|....|....|....|
  Ref       ACGCCTCGTGCGGGCCCTTCGGCTACTGCGACGGCATCGGCGCCACGGCGACTCCGACGTGCAAGTGCCTCGACGGCTTC
  Swarna    ACGCCTCGTGCGGGCCATTCGGCTACTGCGACGGCATCGGCGCCACGGCGACTCCGACGTGCAAGTGCCTCGACGGCTTC
  RP        ACGCCTCGTGCGGGCCATTCGGCTACTGCGACGGCATCGGCGCCACGGCGACTCCGACGTGCAAGTGCCTCGACGGCTTC

                     890       900       910       920       930       940       950       960
            ....|....|....|....|....|....|....|....|....|....|....|....|....|....|....|....|
  Ref       GTCCCCGTCGACAGCAGCCACGACGTCTCGAGAGGTTGCCGGAGGAAGGAGGAGGAGGTGGGATGCGTCGGCAGGCGGCG
  Swarna    GTCCCAGTTGACGGCGGCCACGACGTCTCGAGAGGTTGCCAGAGGAAGGAGGAGGAGGTGGGATGCGTCGGC-------G
  RP        GTCCCCGTTGACGGAAGCCACGACGTCTCGAGAGGTTGCCGGAGGAAGGAGGAGGAGGTGGGATGCGTCGGC-GGCGGCG

                     970       980       990      1000      1010      1020      1030      1040
            ....|....|....|....|....|....|....|....|....|....|....|....|....|....|....|....|
  Ref       GCGGCGATGGCTTCTTGACCATGCCGAGCATGAGGACACCGGACAAGTTCTTGTACGTGAGGAACAGAAGCTTCGATCAG
  Swarna    GCGGAGATGGCTTCTTGACCTTGCCGAGCATGAGGACGCCGGACAAGTTCTTGTACGTGAGGAACAGAAGCTTCGATCAG
  RP        GCGGAGATGGCTTCTTGACCTTGCCGAGCATGAAGACGCCGGACAAGTTCTTGTACGTGAGGAACAGAAGCTTCGATCAG

                    1050      1060      1070      1080      1090      1100      1110      1120
            ....|....|....|....|....|....|....|....|....|....|....|....|....|....|....|....|
  Ref       TGCACCGCAGAGTGCAGCCGCAACTGCTCCTGCACGGCTTATGCTTATGCAATCCTGAATAATGCCGATGCGACGGAGGA
  Swarna    TGCACCGCAGAGTGCAGCCGCAACTGCTACTGCACGGCTTATGCTTATGCAATCCTGAATAACGCCGATGCGACGGAGGA
  RP        TGCACCGCAGAGTGCAGCCGCAACTGCTCCTGCACGGCTTATGCTTATGCACTCCTGAATAATGCCGATGCGACGGAGGA

                    1130      1140      1150      1160      1170      1180      1190      1200
            ....|....|....|....|....|....|....|....|....|....|....|....|....|....|....|....|
  Ref       CCGGTCCAGGTGCTTGGTTTGGATGGGGGAGCTCGTCGACACGGGCAAGTTCAGCGATGGTGCCGGCGGCGAGAACCTCT
  Swarna    CCGGTCCAGGTGCTTGGTTTGGATGGGGGAGCTCGTCGACACGGGCAAGTTCAGCGATGGTGCCGGCGGCGAGAACCTCT
  RP        CCGGTCCAGGTGCTTGGTTTGGATGGGGGAGCTCGTCGACACGGGCAAGTTCAGCGATGGTGCCGGCGGCGAGAACCTCT

                    1210      1220      1230      1240      1250      1260      1270      1280
            ....|....|....|....|....|....|....|....|....|....|....|....|....|....|....|....|
  Ref       ACCTCCGGATTCCTGGCTCACGAGGCATGTATTTCGATAACCTTTATGGTGTTCTCTACTGGTAGTAGAAATGATTTCTT
  Swarna    ACCTCCGGATTCCTGGCTCACGAGGCATGTATTTCGATAACCTTTACGGTGTTCTCTACTGGTTGTAGAAATGATTTCTG
  RP        ACCTCCGGATTCCTGGCTCACGAGGCATGTATTTAGATAACCTTTATGGTGTTCTCTACTGGTAGTAGAAATGATTTCTC

                    1290      1300      1310      1320      1330      1340      1350      1360
            ....|....|....|....|....|....|....|....|....|....|....|....|....|....|....|....|
  Ref       GGATATTTTCTTTATTCTGGACTGTATAATAATATGATTAATTCGCTTGATATGTAATGTGATTAATGATTTCTGCAGCT
  Swarna    GGATATTTTCTTTATTCTGGACTGTATAATAATATGATTAATTCGCCTGATATGTAATGTGATTAATGATTTCTGCAGGT
  RP        GGATATTTTCTTTATTCTGGACTGTATAATAATATGATTAATTCGCTTGATATGTAATGTGATTAATGATTTCTGCAGCT

                    1370      1380      1390      1400      1410      1420      1430      1440
            ....|....|....|....|....|....|....|....|....|....|....|....|....|....|....|....|
  Ref       AATAACAAGACGAAAAGTACTGTTCTGAAGATTGTACTGCCGGTTGCGGCTGGTCTTCTGCTGATACTTGGAGGCATATG
  Swarna    AATAACAAGATGAAAAGTACTGTGCTGAAGATTGTACTGCCGGTTGTGGCTGGTCTTCTGCTGATACTAGGAGGCATATG
  RP        AATAACAAGATGAAAAGTACTGTACTGAAGATTGTACTGCCGGTTGCGGCTGGTCTTCTGCTGATACTTGGAGGCATATG

                    1450      1460      1470      1480      1490      1500      1510      1520
            ....|....|....|....|....|....|....|....|....|....|....|....|....|....|....|....|
  Ref       TCTTGTACGCAAATCAAGAGGTAGAGTGTAAAAAACATTACTTTGCTCTTTGATGAGATATTTCACTTGTATGAAACAAT
  Swarna    TCTTGTACGCAAATCAAGAGGTAGAGTGTAAAAAACATTACTTTGCTCTTTGATGAGATATTTCACTTGTATGAAACAAT
  RP        TCTTGTACGCAAATCAAGAGGTAGAGTGTAAAAAACATTACTTTGCTCTTTGATGAGATATTTCACTTGTATGAAACAAT

                    1530      1540      1550      1560      1570 1580      1590          1600
            ....|....|....|....|....|....|....|....|....|....|....|....|....|....|....|....|
  Ref       AATGATGATACAGAAGCGTTCCTTTCAGGCAATCAACCAAGCAAGAAAGTTCAGAGCAAATATCCATTTCAACACATGAA
  Swarna    AATGATGATACAGAGGCGTTCCTTTCAGGCAATCAACCAAGCAAGAAAGTTCAGAGCAAATATCCATTTCAACACATGAA
  RP        AACGATGATACAGAGGTGTTCCTTTCAGGCAATCAACCAAACAAGAAAGTTCAGAGCAAATATCCATTTCAACACATGAA

                    1610      1620      1630      1640      1650      1660      1670      1680
            ....|....|....|....|....|....|....|....|....|....|....|....|....|....|....|....|
  Ref       TGATTCAAACGAAGTTGGGAGCGAAAATGTAGAACTTTCTTCTGTTGACTTGGATTCTGTCCTCACTGCAACAAACAATT
```

```
Swarna  TGATTCAAACGAAGTTGGGAGCGAAAATGTAGAACTTTCTTCTGTTGACTTGGATTCTGTCCTCACTGCAACAAACAATT
RP      TGATTCAAACGAAGTTGGGAGCGAAAATGTAGAACTTTCTTCTGTTGACTTGGATTCTGTCCTCACTGCAACAAACAATT

            1690      1700      1710      1720      1730      1740      1750      1760
        ....|....|....|....|....|....|....|....|....|....|....|....|....|....|....|....|
Ref     TCTCCGATTACAACTTGCTTGGAAAAGGAGGTTTTGGAAAAGTTTACAAGGTAATAATGATAAGTCTTAGCTTGATTTTT
Swarna  TCTCCGATTACAACTTGCTTGGAAAAGGAGGTTTTGGAAAAGTTTACAAGGTAATAATGATAAGTCTTAGCTTGATTTTT
RP      TCTCCGATTACAACTTGCTTGGAAAAGGAGGTTTTGGAAAAGTTTACAAGGTAATAATGATATGTCTTAGCTTGATTTTT

            1770      1780      1790      1800      1810      1820      1830      1840
        ....|....|....|....|....|....|....|....|....|....|....|....|....|....|....|....|
Ref     TAGTTAAAGATATATGATATGTTATAGCTTATCTCTTGTTTAATCAAAGAATGGTGCAATATATATTTAGGGAGTTTTGG
Swarna  GAGTTAAAGATATATGATATGTTATAGCTTATCTCTTGTTTAATCAAAGAATGGTGCATTATATATTTAGGGAGTTTTGG
RP      GAGTTAAAGATATATGATATGTTATAGCTTATCTCTTGTTTAATCAAAGAATGGTGCAATATATATTTAGGGAGTTTTGG

            1850      1860      1870      1880      1890      1900      1910      1920
        ....|....|....|....|....|....|....|....|....|....|....|....|....|....|....|....|
Ref     AGGGTGGCATAGAAGTTGCTGTCAAGAGGCTTAGCAAGGGTTCAGGGCAAGGTGTTGAGGAGTTCAGAAATGAAGTGGTT
Swarna  AGGGTGGCATAGAAGTTGCTGTCAAGAGGCTTAGCAAGGGTTCAGGGCAAGGTGTTGAGGAGTTCAGAAATGAAGTGGTT
RP      AGGGTGGCATAGAAGTTGCTGTCAAGAGGCTTAGCAAGGGTTCAGGGCAAGGTGTTGAGGAGTTCAGAAATGAAGTGGTT

            1930      1940      1950      1960      1970      1980      1990      2000
        ....|....|....|....|....|....|....|....|....|....|....|....|....|....|....|....|
Ref     CTGATTGCTAAATTACAACACAGAAACTTGGTTAGGCTTCTTGGTTGCTGCATTCATGAAGACGAAAAGTTACTCATCTA
Swarna  CTGATTGCTAAATTACAACACAGAAACTTGGTTAGGCTTCTTGGTTGCTGCATTCATGAAGACGAAAAGTTACTCATCTA
RP      CTGATTGCTAAATTACAACACAGAAACTTGGTTAGGCTTCTTGGTTGCTGCATTCATGAAGACGAAAAGTTACTCATCTA

            2010      2020      2030      2040      2050      2060      2070      2080
        ....|....|....|....|....|....|....|....|....|....|....|....|....|....|....|....|
Ref     CGAATACTTACCAAACAGAAGCTTGGATGCCTTCCTTTTTGGTACGTTATTTCTGACTTATGATCTCTGACAATAAACTG
Swarna  CGAATACTTACCAAACAGAAGCTTGGATGCCTTCCTTTTTGGTACGTTATTTCTGACTTATGATCTCTGACAATAAACTG
RP      CGAATACTTACCAAACAGAAGCTTGGATGCCTTCCTTTTTGGTACGTTGTTTCTGACTTATGATCTCATATATTAA-CTG

            2090      2100      2110      2120      2130      2140      2150      2160
        ....|....|....|....|....|....|....|....|....|....|....|....|....|....|....|....|
Ref     AAGGGATATTGATGATGTCTGACAATAAATTTCATTCCACATTGATCTTGGAAGATGCTAACAGAAAAAACACGCTTGAC
Swarna  AAGGGATATTGATGATGTCTGACAATAAATTTCATTCCACATTGATCTTGGAAGATGCTAACAGAAAAAACACGCTTGAC
RP      AAGGGATATTGATGATGTCTGACAATAAATTTCATTCCACATTGATCTTGGAAGATGCTAACAGAAAAAACACGCTTGAC

            2170      2180      2190      2200      2210      2220      2230      2240
        ....|....|....|....|....|....|....|....|....|....|....|....|....|....|....|....|
Ref     TGGCCGACAAGGTTCAAGATAATTAAAGGAGTAGCAAGAGGTCTTCTTTATCTTCACCAGGATTCAAGATTAACAATAAT
Swarna  TGGCCGACAAGGTTCAAGATAATTAAAGGAGTAGCAAGAGGTCTTCTTTATCTTCACCAGGATTCAAGATTAACAATAAT
RP      TGGCCGACAAGGTTCAAGATAATTAAAGGAGTAGCAAGAGGTCTTCTTTATCTTCACCAGGATTCAAGATTAACAATAAT

            2250      2260      2270      2280      2290      2300      2310      2320
        ....|....|....|....|....|....|....|....|....|....|....|....|....|....|....|....|
Ref     CCATAGAGATCTAAAGACAAGCAACATTTTGTTGGACACAGAAATGAGTCCTAAAATATCAGATTTCGGCATGGCAAGGA
Swarna  CCATAGAGATCTAAAGACAAGCAACATTTTGTTGGACACAGAAATGAGTCCTAAAATATCAGATTTCGGCATGGCAAGGA
RP      CCATAGAGATCTAAAGACAAGCAACATTTTGTTGGACACAGAAATGAGTCCTAAAATATCAGATTTCGGCATGGCAAGGA

            2330      2340      2350      2360      2370      2380      2390      2400
        ....|....|....|....|....|....|....|....|....|....|....|....|....|....|....|....|
Ref     TCTTTGGTGGAAATGAGCAACAAGCTAATACCACCAGGGTTGTCGGCACATAGTAAGCAGTTCATACTTCATATATTAGC
Swarna  TCTTTGGTGGAAATGAGCAACAAGCTAATACCACCAGGGTTGTCGGCACATAGTAAGCAGTTCTTACTTTATATTTGAGG
RP      TCTTTGGTGGAAATGAGCAACAAGCTAATGCCTCCAGGGTTGTCGGCACCTAGTATGCAGTTCATAGTTCATATATTAGC

            2410      2420      2430      2440      2450      2460      2470      2480
        ....|....|....|....|....|....|....|....|....|....|....|....|....|....|....|....|
Ref     TAATGTTCAACGTGCAAACTATTTACCCTAATTCAAACTGCATATTGTTTCCTGAATTATTTAAACAGTGGTTACATGTC
Swarna  TAATGTTCAAAGTGCAAACTATTTACCCTAATTCAAACTGCATATTGTTTCCTGAATTATTTAAACAGTGGTTACATGTT
RP      TAATGTCCA-CGTGCAACCTATCTACCCTAATTCAAACTGCATATTGTTTCGTAAATAATGTAAACAGTGGTTACATGTC

            2490      2500      2510      2520      2530      2540      2550      2560
        ....|....|....|....|....|....|....|....|....|....|....|....|....|....|....|....|
Ref    T-CCTGAATATGCTTTGGATGGCTATTTTTCCGTCAAGTCTGACACCTATAGTTTTGGTGTCATACTGTTGGAAGTTGTG
```

```
Swarna  TTCCTGAATATGCTTTGGATGGCTATTTTTCCGTCAAGTCTGACACCTATAGTTTTGGTGTCATACTATTGGAAGTTTTG
RP      T-CCTGAATATGCTTTGGATGGCTATTTTTCGGTCAAGTCTGACACCTATAGTTCTGGAGTCATACTATTGGAAGTTGTG

                2570      2580      2590      2600      2610      2620      2630      2640
        ....|....|....|....|....|....|....|....|....|....|....|....|....|....|....|....|
Ref     AGTGGCTTAAAGATCAGTTCAGCCCATCTCAAAGTGGACTGTTCAAACCTTATAGCCTATGTAAGTATAGCACAACGCTC
Swarna  AGTGGCTTAAAGATGAGTTCAGCCCATCTCAAAGTGGACTGTTCAAACCTTATAGCCTATGTAAGTATAGCACAACGCTC
RP      AGTGGCTTAAAGATCAGTTCAGCCCATCTCAAAGTGGACTGTTCAAACCTTATAGCATATGTAAGTATAGCACAACGCTC

                2650      2660      2670      2680      2690      2700      2710      2720
        ....|....|....|....|....|....|....|....|....|....|....|....|....|....|....|....|
Ref     AAATTTCTGCAAGTCAAACTAAATGTAATGAGTTGAGATATCATAACTTGATTTGTTTCTTCAGGCATGGAGTTTATGGA
Swarna  AAATTTCTGCAAGTCAAACTAAATGTAATGAGTTGAGATATCATAACTTGATTTGTTTCTTCAGGCATGGAGCTTATGGA
RP      AAATTTCTGCAAGTCAAACTAAATGTAATGAGTTGAGATATCATAACTTGATTTGTCTCCTCAGGCATGGAGCTTATGGA

                2730      2740      2750      2760      2770      2780      2790      2800
        ....|....|....|....|....|....|....|....|....|....|....|....|....|....|....|....|
Ref     AAGATGGAAATGCAAGGGATTTCGTAGACTCATCCATTGTGGAGAGCTGTCCACTTCATGAAGTTCTACGATGTATACAT
Swarna  AAGATGGAAATGCAAGGGATTTCGTAGACTCATCCATTGTGTTGAGCTGTCCACTTCATGAAGTTCTACGATGTATACAT
RP      AAGATGGAAATGCAAGGGATTTCGTAGACTCATCCATTGTGGAGAGCTGTCCACTTCATGAAGTTCTACGATGTATACAT

                2810      2820      2830      2840      2850      2860      2870      2880
        ....|....|....|....|....|....|....|....|....|....|....|....|....|....|....|....|
Ref     TTAGGACTCCTTTGCATTCAAGATCAACCAAGTGCTAGGCCACTGATGTCATCCATTGTGTTCATGCTGGAGAACGAAAC
Swarna  TTAGGACTCCTTTGCATTCAAGATCAACCAAGTGATAGGCCACTGATGTCATCCATTGTGTTCATGCTGGAGAACGAAAT
RP      TTAGGACTCCTTTGCATTCAAGATCAACCAAGTGCTAGGCCACTGATGTCATCCATTGTGTTCATGCTGGAGAACGAAAC

                2890      2900      2910      2920      2930      2940      2950      2960
        ....|....|....|....|....|....|....|....|....|....|....|....|....|....|....|....|
Ref     TGCCGTGCTTCCTGCTCCAAAGGAGCCTATATATTTCACAAGGAGGGAGTACGGGACAGACGAAGACACGAGAGATAGCA
Swarna  TGCCGTGCTTCCTGCTCCAGAGGAGCCTATATATTTCACAAGGAGGGAGTACGGGACAGACGAAGACACGAGAGATAGCA
RP      TGCCGTGCTTCCTGCTCCACAGGAGCCTATATATTTCACAAGGAGGGAGTACGGGACAGACGAAGACACGAGAGATAGCA

                2970      2980      2990      3000      3010      3020      3030      3040
        ....|....|....|....|....|....|....|....|....|....|....|....|....|....|....|....|
Ref     TGAGATCAAGATCTCTGAACCACATGAGTAAAACAGCAGAAGATGGGCGTTAAATGGTTAATTCCGCTGTACAGATCCCA
Swarna  TGAGATCAAGATCTCTGAACCACATGAGTATAACAGCAGAAGATGGGCGTTAAATGGTTAATTCCGCTGTACAGATCCCA
RP      TGAGATCAAGATCTCTGAACCACATGAGTATAACAGCAGAAGATGGGCGTTAAATGGTTAATTCCGCTGTACAGATCCCA

                3050      3060      3070      3080      3090      3100      3110      3120
        ....|....|....|....|....|....|....|....|....|....|....|....|....|....|....|....|
Ref     ATTTTTTAAAACTGTTATTCCAATTGAACAATACCATTACATTGTTTGATTTAAAAAGGTTCTACTGGATACTACATTAT
Swarna  ATTTTTTAAAACTGTTATTCCAATTGAACAATACCATTACATTGTTTGATTTAAAAAGGTTCTACTGGATACTACATTAT
RP      ATTTTTTAAAACTGTTGTTCCAATTGAACAATACCATTACATTGTTTGATTTAAAAAGGTTCTACTGGATACTACATTAT

                3130      3140      3150      3160      3170      3180      3190      3200
        ....|....|....|....|....|....|....|....|....|....|....|....|....|....|....|....|
Ref     TCACTGGTTGGCTTGCGTTTATAAAAACTCTCTTTAGTTTTTTTATATAAATGTAAA-CATTACTCCCTCCTTAGATCT
Swarna  TCACTGGTTGGCTTGCGTTTATAAAAACTCTCTTTAGTTTTTTT-ATATAAATGTAAAACATTACTCCCTCCTTAGATCT
RP      TCACTGGTTGGCTTGCGTTTATAAAAACTCTCTTTAGTTTTTTTATATAAATGTAAAACATTACTCCCTC---AGATCT

                3210      3220      3230      3240      3250      3260      3270      3280
        ....|....|....|....|....|....|....|....|....|....|....|....|....|....|....|....|
Ref     TTATAAGGCACATTTTGATTTAAAAATGTCAAGCTTTATAAACTTTGACCAACAATTAGCGGATGGCATATCTCGTAGGA
Swarna  TTATAAGGCACATTTTGATTTAAAAATGTCAAGCTTTATAAACTTTGACCAACAATTAGCGGATGGCATATCTCGTAGGA
RP      TTATAAGGCACATTTTGATTTAAAAATGTCAAGCTTTATAAACTTTGACCAACAATTAGCGGATGGCATATCTCGTAGGA

                3290      3300      3310      3320      3330      3340      3350      3360
        ....|....|....|....|....|....|....|....|....|....|....|....|....|....|....|....|
Ref     TGTTTATGTAATGACGTGACTAATAAAATAAAATTCCTGATACACTATCAATTAAATTTAACAAACTAAAATGGCAATCC
Swarna  TGTTTATGTAATGATGTGACTAATAAAATAAAATTCCTGATACACTATCAATTAAATTTAACAAACTAAAACGGCAATCC
RP      TGTTTATGTAATGATGTGACTAATAAAATAAAATTCCTGATACACTATCAATTAAATTTAACAAACTAAAACAACAATCC

                3370      3380      3390      3400      3410      3420      3430      3440
        ....|....|....|....|....|....|....|....|....|....|....|....|....|....|....|....|
```

```
Ref       AATTGTGCTGAAATAAATAAAGTATTTGGCAAATTATGAGCTAAATAACAAAGCTAACTTGGGTTTATTTCCACGTTCTA
Swarna    AATTGTGCTGAAATAAATAAAGTATTCGGCAAATTATGAGCTAAATAACAAAGCTAACTTGGGTT-ATTTCCACGTTCTA
RP        AATTGTGCTGAAATAAATAAAGTATTCGGCAAATTATGAGCTCAATAACAAAGCTAACTTGGGTT-ATTTCCACGTTCAC


          ....|
Ref       CCTCC
Swarna    CCTCC
RP        TCCCA
```

Promoter+5′UTR region | Exons | Introns | 3′UTR region | 3′UTR downstream region
