# Supplementary material for: Identification and Functional Characterization of Two Major Loci Associated with Resistance against Brown Planthoppers (Nilaparvata lugens (Stål)) Derived from Oryza nivara
Source: Genes (Basel). 2023 Nov 11;14(11):2066. doi: 10.3390/genes14112066 (PMC10671472; doi:10.3390/genes14112066)
Supplement: Supplementary file 1 [file genes-14-02066-s001.zip › Tables S1, S3-S5.pdf]

Supplemetary Table S1. Summarization of the QTLs that were identified in the backcross introgression mapping population of Swarna × RPbio4918-230S for BPH resistance, employing QTL IciMapping V 4.1.

Trait name	QTL	Chr.	Left Marker	Right Marker	LOD	PVE (%)	Additive Effect
Damage score	*bph 39*	4	RM8213	RM5953	26.50	20.96	2.33
Damage score	*bph 40*	4	RM5953	R4M17	27.02	22.10	2.50

Supplemetary Table S3. Five overlapping primers designed to cover the whole of candidate gene

Primer	Length	Tm	GC%	Amplicon length
TTCTATGCGTGAACATCAGCT	21	57.75	42.86	1108
CTCTTCTTGATCGGGGTGTTG	21	58.65	52.38	
TGATGACAAGCTTACACCTGC	21	58.57	47.62	1036
AACTTGTCCGGTGTCCTCATG	21	60.27	52.38	
ATCCAGACCAACACCAGCTT	20	59.23	50.0	1035
ACATTTTCGCTCCCAACTTCG	21	59.46	47.62	
TCTGAAGATTGTACTGCCGGT	21	59.10	47.62	1001
TGAACTGCTTACTATGTGCCG	21	58.38	47.62	
TCTTGGTTGCTGCATTCATGA	21	58.48	42.86	1529
GGAGACCAGGACCACTTTTCT	21	59.30	52.38	

Supplementary Table S4 : In-Silico analysis for the variations at the Cis-regulatory motifs in the form of SNPs at the promoter/ regulatory regions of the differentially expressed OsSTPKR gene in RPbio4918-230(S) vs SWARNA.

Genotype	Cis-acting motif in 5′ UTR/regulatory region of RP	Location	Type of strand	Signal sequence in RP	Function of motif	Sequence variation observed in the domain of Swarna	Reference
RPbio4918-230(S)	DOFCOREZM	1	Sense	AA**A**G	Core site required for binding of Dof proteins in maize. Dof1 enhances transcription from the promoters of both cytosolic orthophosphate kinase (CyPPDK) and a non-photosynthetic PEPC gene.	AA**G**G	[42]
	DOFCOREZM	39	Antisense	CTT**T**	Core site required for binding of Dof proteins in maize. Dof1 enhances transcription from the promoters of both cytosolic orthophosphate kinase (CyPPDK) and a non-photosynthetic PEPC gene.	CTT**C**	[43]
	ACGTATERD1	260	Sense	A**C**GT	Etiolation-induced expression of erd1 (early responsive to dehydration) in Arabidopsis.	A**A**GT	[44]
	ASF1MOTIFCAMV	261	Antisense	**C**GTC**A**	TGACG motifs are found in many promoters and are involved in transcriptional activation of several genes by auxin and/or salicylic acid. NPR1 disease resistance protein is a novel cofactor that confers redox regulation of DNA binding activity	**A**GTC**G**	[45,46].

					to the basic domain/leucine zipper transcription factor TGA1.		
	WRKY71OS	262	Antisense	GTC**A**	Binding site of rice WRKY71, a transcriptional repressor of the gibberellin signaling pathway. Parsley (*Petroselinum crispum*) WRKY proteins bind specifically to TGAC-containing W box elements within the Pathogenesis-Related Class10 (PR-10) genes.	GTC**G**	[47,48].
	CACTFTPPCA1	348	Antisense	**T**GAA	Tetranucleotide (CACT) is a key component of Mem1 (mesophyll DE expression module 1) found in the cis-regulatory element in the DE distal region of the phosphoenolpyruvate carboxylase (ppcA1).	**G**GAA	[49]
	POLLEN1LELAT52	399	Antisense	TTTC**T**	Required for pollen specific expression; Also found in the promoter of tomato endo-beta-mannanase gene (*LeMAN5*) gene.	TTTC**C**	[50]
	EBOXBNNAPA	498	Antisense	CA**T**C**T**G	Cis-acting elements recognized by R2R3-MYB, BZIP, and BHLH factors control light-responsive and tissue-specific activation of phenylpropanoid biosynthesis genes.	CA**C**C**C**G	[51]
	MYCCONSENSUSAT	498	Antisense	CA**T**C**T**G	MYC recognition site found in the promoters of the dehydration-responsive gene rd22 and many other genes.	CA**C**C**C**G	[52,53]

	ACGTCBOX	504	Sense	GACGTC	Plant bZIP Proteins gather at ACGT elements. The rice bZIP transcriptional activator RITA-1 is highly expressed during seed development	GACCTC	[54,55].
	ACGTATERD1	505	Sense	ACGT	Etiolation-induced expression of erd1 (early responsive to dehydration) in Arabidopsis.	ACCT	[43]
	ARR1AT	525	Antisense	AATCG	"ARR1-binding element" found in Arabidopsis; AGATT is found in the promoter of rice non-symbiotic haemoglobin-2 (NSHB) gene.	AACGG	[55]
	NODCON2GM	533	Antisense	AAGAG	One of two putative nodulin consensus sequences	ACGAG	[56]
	OSE2ROOTNODULE	533	Antisense	AAGAG	One of the consensus sequence motifs of organ-specific elements (OSE) characteristic of the promoters activated in infected cells of root nodules	ACGAG	[57].
	CGACGOSAMY3	580	Antisense	CGTCG	"CGACG element" found in the GC-rich regions of the rice. Function as a coupling element for the G box element.	CGGCG	[58]
	RHERPATEXPA7	582	Antisense	TCGTG	Root Hair-specific cis-Elements; conserved among the Arabidopsis thaliana.	GCGTG	[59]
	HEXAMERATH4	663	Sense	CCGTCG	Hexamer motif of Arabidopsis thaliana (A.t.) at histone H4 promoter.	CCGTCA	[60]
	CGACGOSAMY3	664	Antisense	CGTCG	"CGACG element" found in the GC-rich regions of the rice. Function as a coupling element for the G box	CGACG	[58].

					element.		
	HEXAMERATH4	669	Sense	CCGTCG	Hexamer motif of Arabidopsis thaliana (A.t.) at histone H4 promoter.	CCGACG	[60]
	CGACGOSAMY3	670	Antisense	CGTCG	"CGACG element" found in the GC-rich regions of the rice. Function as a coupling element for the G box element.	CGACG	[58]
	LTRECOREATCOR15	671	Antisense	GTCGG	Core of low temperature responsive element (LTRE) of cor15a gene.	GACGG	[61]
	DRECRTCOREAT	671	Antisense	GTCGG	Core motif of DRE/CRT (dehydration-responsive element/C-repeat) cis-acting element found in many genes.	GACGG	[62]
	CBFHV	671	Antisense	GTCGGC	CBFs are also known as dehydration-responsive element (DRE) binding proteins (DREBs). Binding site of barley (H.v.) CBF1.	GACGG	[63]
	HEXAMERATH4	680	Sense	CCGTCG	Hexamer motif of Arabidopsis thaliana (A.t.) at histone H4 promoter.	CCGCCC	[60]
	CGACGOSAMY3	681	Antisense	CGTCG	"CGACG element" found in the GC-rich regions of the rice. Function as a coupling element for the G box element.	CGCCC	[58]
	CBFHV	682	Antisense	GTCGAT	CBFs are also known as dehydration-responsive element (DRE) binding proteins (DREBs). Binding site of barley (H.v.) CBF1.	GCCCAT	[63]
	TGACGTVMAMY	743	Antisense	ACGTCA	Required for high level expression of alpha-Amylase in the cotyledons of	ACGTCG	[64]

					the germinated seeds.		
	HEXMOTIFTAH3H4	743	Sense	ACGTC**A**	"hexamer motif" in type 1 element may play important roles in regulation of replication- dependent but not of replication-independent expression of the wheat histone H3 gene; Rice OBF1-homodimer-binding site.	ACGTC**G**	[65]
	ASF1MOTIFCAMV	744	Antisense	CGTC**A**	TGACG motifs are found in many promoters and are involved in transcriptional activation of several genes by auxin and/or salicylic acid. NPR1 disease resistance protein is a novel cofactor that confers redox regulation of DNA binding activity to the basic domain/leucine zipper transcription factor TGA1.	CGTC**G**	[44,45]
	WRKY71OS	745	Antisense	GTC**A**	Binding site of rice WRKY71, a transcriptional repressor of the gibberellin signaling pathway. Parsley (Petroselinum crispum) WRKY proteins bind specifically to TGAC-containing W box elements within the Pathogenesis-Related Class10 (PR-10) genes.	GTC**G**	[46,47]

	UPRMOTIFIIAT	875	Sense	CC**C**GTT GACGG**A** **A**GCCAC G	"Motif II" in the conserved UPR (unfolded protein response) DE cis-acting element in Arabidopsis genes coding for SAR1B, HSP-90, SBR-like, Ca-ATPase 4, CNX1, PDI etc.,.	CC**A**GTT GACGG**C** **G**GCCAC G	[66]

Supplementary Table S5: In-Silico analysis for the variations at the Cis-regulatory motifs in the form of SNPs at the promoter/ regulatory regions of the differentially expressed OsSTPKR gene in SWARNA vs RPbio4918-230(S).

Genotype	Cis-acting motif in 5′ UTR/regulatory region of Swarna	Location	Type of strand	Signal sequence in Swarna	Function of motif	Sequence variation observed in the domain of RP	Reference
Swarna	DOFCOREZM	1	Sense	AAG	Core site required for binding of Dof proteins in maize. Dof1 enhances transcription from the promoters of both cytosolic orthophosphate kinase (CyPPDK) and a non-photosynthetic PEPC gene.	AAA	[42]
	GCCCORE	4	Antisense	GGCGGC	Core of GCC-box found in many pathogen-responsive genes such as PDF1.2, Thi2.1, and PR4.	AGTTCC	[67,68]
	LTRECOREATCOR15	65	Sense	CCGAC	Core of low temperature responsive element (LTRE) of cor15a gene in Arabidopsis (A.t.)	CCAAC	[61]
	PRECONSCRHSP70A	65	Sense	CCGACGA GCTCAACT	Consensus sequence of PRE (plastid response element) in the promoters of HSP70A in Chlamydomonas; Involved in induction of HSP70A gene by both MgProto and light.	CCAACGA GCTCAAC T	[69]
	CGACGOSAMY3	66	Sense	CGACG	"CGACG element" found in the GC-rich regions of the rice (O.s.) Amy3D and Amy3E amylase genes. May function as a coupling element for the G box	CAACG	[58]

					element.		
	TBOXATGAPB	262	Antisense	CAAAGT	"Tbox" found in the Arabidopsis thaliana (A.T.) GAPB gene promoter. GAPB encodes the B subunit of chloroplast glyceraldehyde-3-phosphate dehydrogenase (GADPH).	CAACGT	[70]
	DOFCOREZM	263	Sense	AAAG	Core site required for binding of Dof proteins in maize. Dof1 enhances transcription from the promoters of both cytosolic orthophosphate kinase (CyPPDK) and a non-photosynthetic PEPC gene.	AACG	[42]
	GCCCORE	269	Sense	GCCGCC	Core of GCC-box found in many pathogen-responsive genes such as PDF1.2, Thi2.1, and PR4.	ACCGCC	[67,68]
	BP5OSWX	394	Sense	CAACGTG	OsBP-5 (a MYC protein) binding site in Waxy promoter.	CAACGTA	[71]
	T/GBOXATPIN2	395	Sense	AACGTG	"T/G-box" found in tomato proteinase inhibitor II (pin2) and leucine aminopeptidase (LAP) genes; Involved in jasmonate (JA) induction of these genes; bHLH-Leu zipper JAMYC2 and JAMYC10 proteins specifically recognize this motif	AACGTA	[72]
	RHERPATEXPA7	396	Antisense	ACGTGA	"Right part of RHEs (Root Hair-specific cis-Elements)"	ACGTAA	[59]

					conserved among the Arabidopsis thaliana A7 (AtEXPA7) orthologous (and paralogous) genes from diverse angiosperm species with different hair distribution patterns.		
	ABRELATERD1	396	Sense	ACGT**G**	ABRE-like sequence required for etiolation-induced expression of erd1 (early responsive to dehydration).	ACGT**A**	[43]
	GTGANTG10	398	Sense	GT**G**A	"GTGA motif" found in the promoter of the tobacco (N.t.) late pollen gene g10 which shows homology to pectate lyase and is the putative homologue of the tomato gene lat56.	GT**A**A	[73]
	EECCRCAH1	400	Sense	**G**AGTTTC	"EEC"; Consensus motif of the two enhancer elements, EE-1 and EE-2, both found in the promoter region of the Chlamydomonas-*Cah1* (encoding a periplasmic carbonic anhydrase)	**A**AGTTTC	[74].
	E2F1OSPCNA	403	Antisense	TTTC**C**CGC	"re2f-1" found in the promoter of rice PCNA gene. Involved in transcriptional activation in actively dividing cells and tissue.	TTTC**T**CG C	[75]
	E2FANTRNR	403	Sense	TTTC**C**CGC	Binding site of tobacco and Arabidopsis E2F. Involved in upregulation of the promoter at	TTTC**T**CG C	[76]

					G1/S transition.		
	E2FCONSENSUS	403	Sense	TTTCCCGC	"E2F consensus sequence" of all different E2F-DP-binding motifs that were experimentally verified in plants.	TTTCTCG C	[77]
	BS1EGCCR	406	Antisense	CCCGCT	"BS1 (binding site 1)" found in E. gunnii Cinnamoyl-CoA reductase (CCR) gene promoter. Required for vascular expression.	CTCGCT	[78]
	GTGANTG10	483	Antisense	TCAC	"GTGA motif" found in the promoter of the tobacco (N.t.) late pollen gene g10 which shows homology to pectate lyase and is the putative homologue of the tomato gene lat56.	TCGC	[73]
	GTGANTG10	501	Antisense	TCAC	"GTGA motif" found in the promoter of the tobacco (N.t.) late pollen gene g10 which shows homology to pectate lyase and is the putative homologue of the tomato gene lat56.	CCAT	[73]
	MYBCOREATCYCB1	548	Sense	AACGG	"Myb core" in the sequence is able to activate reporter gene without leading to M-phase-specific expression.	AATCG	[79].
	CGCGBOXAT	588	Sense	GCGCGT	"CGCG box" recognized by AtSR1-6 (Arabidopsis thaliana DE signal-responsive genes); Multiple CGCG elements are found in promoters of many genes.	GGGCGT	[80]

	CANBNNAPA	615	Sense	CCAACAC	Core of "(CA)n element" in storage protein genes. Embryo- and endosperm-specific transcription of napin (storage protein) gene.	TCAACAC	[81]
	ASF1MOTIFCAMV	685	Antisense	CGTCA	TGACG motifs are found in many promoters and are involved in transcriptional activation of several genes by auxin and/or salicylic acid. NPR1 disease resistance protein is a novel cofactor that confers redox regulation of DNA binding activity to the basic domain/leucine zipper transcription factor TGA1.	CGTCG	[44,45]
	WRKY71OS	686	Antisense	GTCA	Binding site of rice WRKY71, a transcriptional repressor of the gibberellin signaling pathway. Parsley (Petroselinum crispum) WRKY proteins bind specifically to TGAC-containing W box elements within the Pathogenesis-Related Class10 (PR-10) genes.	GTCG	[46,47]
	GTGANTG10	687	Antisense	TCAC	"GTGA motif" found in the promoter of the tobacco (N.t.) late pollen gene g10 which shows homology to pectate lyase and is the putative homologue of the tomato gene lat56.	TCGC	[73]

	DRE2COREZMRAB17	689	Sense	ACCGAC	"DRE2" core found in maize (Z.M.) rab17 gene promoter; rab17 is expressed during late embryogenesis, and is induced by ABA.	GCCGTC	[62]
	DRECRTCOREAT	689	Sense	ACCGAC	Core motif of DRE/CRT (dehydration-responsive element/C-repeat) cis-acting element found in many genes in Arabidopsis and in rice. HaDREB2 physically interact with HaHSFA9 in vitro.	GCCGTC	[62]
	CBFHV	689	Sense	ACCGAC	CBFs are known as dehydration-responsive element (DRE) binding proteins (DREBs).	GCCGTC	[82]
	LTRECOREATCOR15	690	Sense	CCGAC	Core of low temperature responsive element (LTRE) of cor15a gene.	CCGTC	[83]
	HEXAMERATH4	691	Antisense	CGACGG	hexamer motif of Arabidopsis thaliana (A.t.) histone H4 promoter.	CGTCGG	[60]
	CGACGOSAMY3	691	Sense	CGACG	"CGACG element" found in the GC-rich regions of the rice (O.s.) Amy3D and Amy3E amylase genes.	CGTCG	[58]
	GCCCORE	700	Sense	GCCGCC	Core of GCC-box found in many pathogen-responsive genes such as PDF1.2, Thi2.1, and PR4.	GCCGTC	[67,68]
	CGACGOSAMY3	765	Antisense	CGTCG	"CGACG element" found in the GC-rich regions of the rice	CGTCA	[58]

					(O.s.) Amy3D and Amy3E amylase genes.		
	CGACGOSAMY3	768	Antisense	CGTCG	"CGACG element" found in the GC-rich regions of the rice (O.s.) Amy3D and Amy3E amylase genes.	CATCG	[58]
	CURECORECR	790	Sense	GTAC	GTAC is the core of a CuRE (copper-response element) found in Cyc6 and Cpx1 genes in Chlamydomonas.	GTTC	[84]
	EBOXBNNAPA	897	Sense	CAGTTG	E-box of napA storage-protein gene of *Brassica napus*.	CCGTTG	[50]
	MYBCORE	897	Sense	CAGTTG	Binding site for all animal MYB and at least two plant MYB proteins ATMYB1 and ATMYB2, both isolated from Arabidopsis. ATMYB2 is involved in regulation of genes that are responsive to water stress in Arabidopsis. A petunia MYB protein (MYB.Ph3) is DE involved in regulation of flavonoid biosynthesis	CCGTTG	[85,86]
	MYB2CONSENSUSAT	897	Antisense	CAGTTG	MYB recognition site found in the promoters of the dehydration-responsive gene rd22 and many other genes	CCGTTG	[51]
	MYCCONSENSUSAT	897	Sense	CAGTTG	MYC recognition site found in the promoters of the dehydration-responsive gene rd22 and many other genes in Arabidopsis.	CCGTTG	[87]

	GCCCORE	905	Antisense	GGCGGC	Core of GCC-box found in many pathogen-responsive genes such as PDF1.2, Thi2.1, and PR4.	GGAAGC	[67,68]

References

[42] S. Yanagisawa, Dof1 and Dof2 transcription factors are associated with expression of multiple genes involved in carbon metabolism in maize, The Plant J. 21 (2000) 281-288.

[43] S.D. Simpson, K. Nakashima, Y. Narusaka, M. Seki, K. Shinozaki, Y.S. Kazuko, two different novel cis-acting elements of erd1, a clpA homologous Arabidopsis gene function in induction by dehydration stress and dark-induced senescence, The Plant J. 33 (2003) 259-270.

[44] C. Després, C. Chubak, A. Rochon, R. Clark, T. Bethune, D. Desveaux, R.F. Pierre, The Arabidopsis NPR1 disease resistance protein is a novel cofactor that confers redox regulation of DNA binding activity to the basic domain/leucine zipper transcription factor TGA1, The Plant Cell 15 (2003) 2181-2191.

[45J. Redman, J. Whitcraft, C. Johnson, J. Arias, Abiotic and biotic stress differentially stimulate as-1 element activity in Arabidopsis, Plant CellRep. 21 (2002) 180-185.

[46] T. Eulgem, J.R. Paul, S. Elmon, H. Klaus, S.E. Imre, Early nuclear events in plant defence signalling: rapid gene activation by WRKY transcription factors, The EMBO J. 18 (1999) 4689-4699.

[47] Z.L. Zhang, Z. Xie, X. Zou, J. Casaretto, H. Tuan-hua David, Q.J. Shen, A rice WRKY gene encodes a transcriptional repressor of the gibberellin signaling pathway in aleurone cells, Plant Physiol. 134 (2004): 1500-1513.

[48] U. Gowik, B. Janet, A. Meryem, U. Schlue, M. Koczor, M. Streubel, P. Westhoff, cis-Regulatory elements for mesophyll-specific gene expression in the C4 plant Flaveria trinervia, the promoter of the C4 phosphoenolpyruvate carboxylase gene, The Plant Cell 16 (2004) 1077-1090.

[49] A.S. Filichkin, M.L. Jeffrey, A. Monteros, P.P. Liu, N. Hiroyuki, A novel endo-β-mannanase gene in tomato LeMAN5 is associated with anther and pollen development, Plant Physiol. 134 (2004) 1080-1087.

[50] U. Hartmann, S. Martin, M. Frank, S. Ralf, B. Weisshaar, Differential combinatorial interactions of cis-acting elements recognized by R2R3-MYB, BZIP, and BHLH factors control light-responsive and tissue-specific activation of phenylpropanoid biosynthesis genes, Plant Mol. Biol. 57 (2005) 155-171.

[51] H. Abe, U. Takeshi, I. Takuya, S. Motoaki, S. Kazuo, Y.S. Kazuko, Arabidopsis AtMYC2 (bHLH) and AtMYB2 (MYB) function as transcriptional activators in abscisic acid signaling, The Plant Cell 15 (2003) 63-78.

[52] V. Chinnusamy, O. Masaru, K. Siddhartha, L. Byeong-ha, H. Xuhui, A. Manu, J. K. Zhu, ICE1: a regulator of cold-induced transcriptome and freezing tolerance in Arabidopsis, Genes & Dev. 17 (2003): 1043-1054.

[53] R. Foster, I. Takeshi, N. H. Chua, Plant bZIP proteins gather at ACGT elements, The FASEB J. (1994) 192-200.

[54] T. Izawa, R. Foster, M. Nakajima, K. Shimamoto, N.H. Chua, The rice bZIP transcriptional activator RITA-1 is highly expressed during seed development, The Plant Cell 6 (1994) 1277-1287.

[55] E.J. Ross, J.M. Stone, C.G. Elowsky, R. Arredondo-Peter, R.V. Klucas, G. Sarath, Activation of the Oryza sativa non-symbiotic haemoglobin-2 promoter by the cytokinin-regulated transcription factor, ARR1, J. Exp.Bot. 55 (2004) 1721-1731.

[56] J. Stougaard, J.E. Jørgensen, T. Christensen, A. Kühle, K.A. Marcker, Interdependence and nodule specificity of cis-acting regulatory elements in the soybean leghemoglobin lbc 3 and N23 gene promoters, Mol. Gener. Genet. 220 (1990) 353-360.

[57] V. Fehlberg, M.F. Vieweg, E.M. Dohmann, N. Hohnjec, A. Pühler, A.M. Perlick, H. Küster, The promoter of the leghaemoglobin gene VfLb29: functional analysis and identification of modules necessary for its activation in the infected cells of root nodules and in the arbuscule-containing cells of mycorrhizal roots, J. Exp. Bot. 56 (2005) 799-806.

[58] Y.S. Hwang, E.E. Karrer, B.R. Thomas, L. Chen, R. L. Rodriguez, Three cis-elements required for rice α-amylase Amy3D expression during sugar starvation, Plant. Mol. Biol. 36 (1998) 331-341.

[59] D.W. Kim, S. H Lee, S.B. Choi, S.K. Won, Y.K. Heo, M. Cho, Y. Park, H.T. Cho, Functional conservation of a root hair cell-specific cis-element in angiosperms with different root hair distribution patterns, The Plant Cell 18 (2006) 2958-2970.

[60] N. Chaubet, F. Martine, C. Bernadette, B. Pierre, G. Claude, Identification of cis-elements regulating the expression of an Arabidopsis histone H4 gene, The Plant. J. 10 (1996) 425-435.

[61] C. Jiang, L. Betty, J. Singh, Requirement of a CCGAC cis-acting element for cold induction of the BN115 gene from winter Brassica napus,Plant. Mol. Biol. 30 (1996) 679-684.

[62] J.G. Dubouzet, Y. Sakuma, Y. Ito, M. Kasuga, E.G. Dubouzet, S. Miura, M. Seki, K. Shinozaki, K. Yamaguchi-Shinozaki, OsDREB genes in rice, Oryza sativa L., encode transcription activators that function in drought-, high-salt-and cold-responsive gene expression, The Plant J. 33 (2003) 751-763.

[63] G.P. Xue, An AP2 domain transcription factor HvCBF1 activates expression of cold-responsive genes in barley through interaction with a (G/a) (C/t) CGAC motif, Biochimi. Biophy. Acta (BBA)-Gene Structure and Expression 1577 (2002) 63-72.

[64] D. Yamauchi, ATGACGT motif in the 5′-upstream region of α-amylase gene from Vigna mungo is a cis-element for expression in cotyledons of germinated seeds, Plant. Cell Physiol. 42 (2001) 635-641.

[65] H. Shimizu, S. Kazuhito, B. Thomas, A. Miyazaki, R. Ozaki, R. Imai, T. Kusano, LIP19, a basic region leucine zipper protein, is a Fos-like molecular switch in the cold signaling of rice plants, Plant. Cell Physiol. 46 (2005) 1623-1634.

[66] M.I. Martínez, M. J. Chrispeels, Genomic analysis of the unfolded protein response in Arabidopsis shows its connection to important cellular processes, The Plant Cell 15 (2003) 561-576.

[67] R.L. Brown, K. Kemal, C. McGrath Ken, J. Maclean Don, and M. Manners John. "A role for the GCC-box in jasmonate-mediatedactivation of the PDF1. 2 gene of Arabidopsis." Plant physiology 132, no. 2 (2003): 1020-1032.

[68] S. Chakravarthy, P.T. Robert, D. D'Ascenzo Mark, R.F. Pierre, Després Charles, and B.M. Gregory. "The tomato transcription factor Pti4 regulates defense-related gene expression via GCC box and non-GCC box cis elements." The Plant Cell 15, no. 12 (2003): 3033-3050.

[69] E.D. von Gromoff, S. Michael, O. Ulrike, and F. B. Christoph. "Identification of a plastid response element that acts as an enhancer within the Chlamydomonas HSP70A promoter." Nucleic acids research 34, no. 17 (2006): 4767-4779.

[70] C.Chan, G.Lankai, and S. Ming-Che. "Promoter analysis of the nuclear gene encoding the chloroplast glyceraldehyde-3-phosphate dehydrogenase B subunit of Arabidopsis thaliana." Plant molecular biology 46, no. 2 (2001): 131-141.

[71] Y. Zhu, C. Xiu-Ling, W. Zong-Yang, and H. Meng-Min. "An interaction between a MYC protein and an EREBP protein is involved in transcriptional regulation of the rice Wx gene." Journal of Biological Chemistry 278, no. 48 (2003): 47803-47811.

[72] M. Boter, O. Ruíz-Rivero, A. Abdeen, and P. Salomé. "Conserved MYC transcription factors play a key role in jasmonate signaling both in tomato and Arabidopsis." Genes & development 18, no. 13 (2004): 1577-1591.

[73] H. J. Rogers, N. Bate, J. Combe, J. Sullivan, J. Sweetman, C. Swan, D. M. Lonsdale, and D. Twell. "Functional analysis of cis- regulatory elements within the promoter of the tobacco late pollen gene g10." Plant molecular biology 45, no. 5 (2001): 577-585.

[74] Kucho K, Yoshioka S, Taniguchi F, Ohyama K, Fukuzawa H. Cis-acting elements and DNA-binding proteins involved in CO2-responsive transcriptional activation of Cah1 encoding a periplasmic carbonic anhydrase in Chlamydomonas reinhardtii. Plant Physiol. 2003 Oct;133(2):783-93. doi: 10.1104/pp.103.026492. PMID: 14555782; PMCID: PMC219052.

[75] S. Kosugi, and Y. Ohashi. "E2F sites that can interact with E2F proteins cloned from rice are required for meristematic tissue-specific expression of rice and tobacco proliferating cell nuclear antigen promoters." The Plant Journal 29, no. 1 (2002): 45-59.

[76] R. Sozzani, , C. Maggio, S. Varotto, S. Canova, C. Bergounioux, D. Albani, and R. Cella. "Interplay between Arabidopsis activating factors E2Fb and E2Fa in cell cycle progression and development." Plant physiology 140, no. 4 (2006): 1355-1366.

[77] K. Vandepoele, , V. Kobe, F. Kobe, L. Hennig, T.S. Beemster Gerrit, W. Gruissem, Y.Van de Peer, D. Inzé, and L. De Veylder. "Genome-wide identification of potential plant E2F target genes." Plant physiology 139, no. 1 (2005): 316-328.

[78] E. Lacombe, J.V. Doorsselaere, B. Wout, A.M. Boudet, and J.G. Pettenati. "Characterization of cis-elements required for vascular expression of the Cinnamoyl CoA Reductase gene and for protein–DNA complex formation." The Plant Journal 23, no. 5 (2000): 663-676.

[79] Planchais, P. Claudette, G. Nathalie, M. Vladimir, I. Dirk, and C. Bergounioux. "Characterization of cis-acting element involved in cell cycle phase-independent activation of Arath; CycB1; 1 transcription and identification of putative regulatory proteins." Plant molecular biology 50, no. 1 (2002): 109-125.

[80] T. Yang, and B. W. Poovaiah. "A calmodulin-binding/CGCG box DNA-binding protein family involved in multiple signaling pathways in plants." Journal of Biological Chemistry 277, no. 47 (2002): 45049-45058.

[81] M. Ellerström, , K. Stålberg, E. Inés, and L. Rask. "Functional dissection of a napin gene promoter: identification of promoter elements required for embryo and endosperm-specific transcription." Plant molecular biology 32, no. 6 (1996): 1019-1027.

[82] J. Svensson, A. M. Ismail, E. T. Palva, and T. J. Close. "Dehydrins. En: Storey KB, Storey JM (eds.). Sensing Signaling and Cell Adaptation." (2002): 155-71.

[83] S.S. Baker, S.W. Kathy, and F. Michael. Thomashow. "The 5′-region of Arabidopsis thaliana cor15a has cis-acting elements that confer cold-, drought-and ABA-regulated gene expression." Plant molecular biology 24, no. 5 (1994): 701-713.

[84] J. Kropat, S. Tottey, R.P. Birkenbihl, D. Nathalie, P. Huijser, and S. Merchant. "A regulator of nutritional copper signaling in Chlamydomonas is an SBP domain protein that recognizes the GTAC core of copper response element." Proceedings of the National Academy of Sciences 102, no. 51 (2005): 18730-18735.

[85] T. Urao, K. Yamaguchi-Shinozaki, S. Urao, and K. Shinozaki. "An Arabidopsis myb homolog is induced by dehydration stress and itsgene product binds to the conserved MYB recognition sequence." The Plant Cell 5, no. 11 (1993): 1529-1539.

[86] R. Solano, C. Nieto, J. Avila, L. Canas, I. Diaz, and J. Paz-Ares. "Dual DNA binding specificity of a petal epidermis-specific MYBtranscription factor (MYB. Ph3) from Petunia hybrida." The EMBO Journal 14, no. 8 (1995): 1773-1784.

[87] P. K.Agarwal, P. Agarwal, M. K. Reddy, and S. K. Sopory. "Role of DREB transcription factors in abiotic and biotic stress tolerance inplants." Plant cell reports 25, no. 12 (2006): 1263-1274.
